# Everybody needs sphingolipids, right! Mining for new drug targets in protozoan sphingolipid biosynthesis

**DOI:** 10.1017/S0031182017001081

**Published:** 2017-06-22

**Authors:** JOHN G. M. MINA, P. W. DENNY

**Affiliations:** Department of Biosciences, Lower Mountjoy, Stockton Road, Durham DH1 3LE, UK

**Keywords:** sphingolipids, ceramide, drug targets, protozoan parasites, apicomplexa, kinetoplastidae

## Abstract

Sphingolipids (SLs) are an integral part of all eukaryotic cellular membranes. In addition, they have indispensable functions as signalling molecules controlling a myriad of cellular events. Disruption of either the *de novo* synthesis or the degradation pathways has been shown to have detrimental effects. The earlier identification of selective inhibitors of fungal SL biosynthesis promised potent broad-spectrum anti-fungal agents, which later encouraged testing some of those agents against protozoan parasites. In this review we focus on the key enzymes of the SL *de novo* biosynthetic pathway in protozoan parasites of the Apicomplexa and Kinetoplastidae, outlining the divergence and interconnection between host and pathogen metabolism. The druggability of the SL biosynthesis is considered, alongside recent technology advances that will enable the dissection and analyses of this pathway in the parasitic protozoa. The future impact of these advances for the development of new therapeutics for both globally threatening and neglected infectious diseases is potentially profound.

## INTRODUCTION

### Protozoan parasites and the global burden of their diseases

Protozoa (kingdom Protista) are single-cell organisms that can be free-living or parasitic in nature (Baron, 1996). Out of more than 50 000 protozoan species that have been described to-date, relatively few have been identified as major contributors to the global burden of human diseases (Kuris, 2012) and animal agriculture (Dubey, 1977). The protozoa represent 19% of all human parasites (83 out of 437 species to-date) and are associated with 30% of parasite-induced human morbidity-mortality (Kuris, 2012).

Of the four groups of infectious protozoa (CDC, 2017), the Mastigophora (flagellates) and Sporozoa contain the Kinetoplastidae and Apicomplexa, respectively. It is to these two phyla that belong many of the causative agents of disease: Mastigophora – the insect vector-borne kinetoplastids *Trypanosoma brucei* (Human African Trypanosomiasis, HAT), *Leishmania* spp. (leishmaniasis, cutaneous and visceral) and *Trypanosoma cruzi* (American trypanosomiasis, Chagas’ disease); Sporozoa – the apicomplexan *Toxoplasma gondii* (toxoplasmosis), *Cryptosporidium* spp. (cryptosporidiosis) and *Eimeria* spp. (coccidiosis in poultry and cattle), *Theileria* spp. (East Coast Fever in cattle) and *Plasmodium* spp., including *Plasmodium falciparum* the causative agent of severe malaria and one of the ‘Big Three’ global infectious diseases alongside HIV and tuberculosis (Torgerson & Macpherson, 2011).

Historically, the diseases caused by some of these parasites have been classified as Neglected Tropical Diseases (NTDs) or Neglected Zoonotic Diseases (King, 2011) and were associated with the classical model of the ‘poverty trap’ covering tropical and sub-tropical regions in Africa, Latin America and the Indian subcontinent (Kuris, 2012). However, with global changes in climate and human demographics and associated practices, the classical models do not promise safe boundaries that might contain and/or stop the further global spread of many of these parasitic diseases (Colwell *et al.* 2011). The problems associated with these pathogens are further aggravated by the lack of effective vaccines (Dumonteil, 2007; Innes *et al.* 2011; McAllister, 2014; Black & Mansfield, 2016) and the paucity of reliable drugs (Zofou *et al.* 2014), in addition to the difficulties of vector or reservoir control (Colwell *et al.* 2011). Therefore, there is a recognized need to find new therapeutic targets in these causative agents in order to develop effective treatment regimens to avoid potentially catastrophic outbreaks, both in terms of human health and economic impact.

This review presents sphingolipid (SL) biosynthesis and ceramide (CER) homoeostasis as a potential gold mine of tractable drug targets for these protozoan parasites.

### State-of-the-art treatment of apicomplexan and kinetoplastid diseases

In general, available treatments for the diseases caused by the Kinetoplastidae and Apicomplexa are outdated (if not historic), with relatively few examples that were introduced recently, toxic and require a long treatment regimen, and therefore close monitoring of patients.

The kinetoplastid pathogens in focus here all cause NTDs and as such there are significant problems with the available drug regimens:

#### *Leishmania* spp

The treatment of leishmaniasis often requires a long course of intravenous pentavalent antimony drugs (e.g. Glucantime and Pentostam), aminosidine (paromomycin) or liposomal amphotericin B (Croft & Coombs, 2003; Center for Food Security and Public Health, 2004; WHO, 2004; Kedzierski *et al.* 2009). The most recent addition was the orally available miltefosine (Sunder *et al.* 2002; Verma & Dey, 2004), originally developed as anti-neoplastic agent. Despite its teratogenic effects (Sunder *et al.* 2002), due to the lack of other effective medications, it has been registered and is now used in India, Colombia, Guatemala and Germany (Soto & Berman, 2006). Other regimens of treatment include Pentamidine (Bray *et al.* 2003), allopurinol, dapsone, fluconazole, itraconazole and ketoconazole. However, to-date all available chemotherapeutic agents suffer from being toxic (Chappuis *et al.* 2007) or inaccessible, both geographically and financially, in endemic areas where public health is under-resourced, poor and underdeveloped. Additionally, the lack of effective vaccines (de Oliveira *et al.* 2009) and the alarming emergence of resistance to these drugs (Croft *et al.* 2006), combined with the short-lived prevention resulting from applying measures such as vector and reservoir host control (WHO, 2004; Figueiredo *et al.* 2012), demand an intensive search for alternative anti-leishmanials to enable effective treatment and control.

#### Trypanosoma brucei

Another compelling example of the shortcomings of available treatments is HAT (Mina *et al.* 2009; Buckner *et al.* 2012), where there is a lack of effective vaccines (Black & Mansfield, 2016) and treatment depends on the stage of the disease. Whilst in the first stage, the drugs used are less toxic, easier to administer and more effective, treatment in the second stage requires drugs that can cross the blood-brain barrier, specifically the arsenates (Gibaud & Jaouen, 2010), making them considerably more toxic and complex to administer (Babokhov *et al.* 2013). Currently, four drugs are registered for HAT treatment and are provided free of charge to endemic countries through a WHO private partnership with Sanofi-Aventis (Pentamidine, melarsoprol and eflornithine) and Bayer AG (suramin) (Schmidt *et al.* 2012). Unfortunately, all of them exhibit a broad range of adverse effects. Moreover, treatment regimens are usually highly restrictive, particularly in the second stage of the disease, requiring hospital-based I.V. treatment with continuous monitoring.

#### Trypanosoma cruzi

Despite their toxic side-effects, nifurtimox and benznidazole are the only licenced drugs available for treatment of Chagas’ disease (Carabarin-Lima *et al.* 2013; Bermudez *et al.* 2016), with the latter being the first choice due to its lower side effects. Also, benznidazole has been implemented in the treatment of women before pregnancy in order to prevent/reduce vertical transmission (Carabarin-Lima *et al.* 2013; Murcia *et al.* 2013). Due to the lack alternatives, efforts have been directed towards implementing different treatment regimens in order to reduce toxicity, e.g. intermittent administration schedules, combination therapy and re-purposing of commercial drugs (Bermudez *et al.* 2016).

Management of apicomplexan infections is also challenging and faces many of the same shortcomings encountered in the treatment of kinetoplastid infections.

#### Toxoplasma gondii

Treatment regimens for toxoplasmosis patients have essentially remained the same since the 1950s (Eyles & Coleman, 1953). They largely depend on the repurposing of antibacterials (sulfadiazine, spiramycin and clindamycin) and antimalarials (pyrimethamine and atovaquone) (Opremcak *et al.* 1992; Andrews *et al.* 2014; Antczak *et al.* 2016) in combination, therapies that target parasite folic acid synthesis, protein synthesis or oxidative phosphorylation (Greif *et al.* 2001; Antczak *et al.* 2016). Most of these chemotherapeutics are not readily bioavailable at the site of infection (e.g. unable to cross the blood-brain barrier); cannot be administered by patients with hypersensitivity to sulphonamides; have suspected teratogenic properties (Montoya & Remington, 2008; Paquet & Yudin, 2013); are threatened by the emergence of resistance (Sims, 2009); or require adjuvant therapies (folinic acid supplement) to minimize toxic side effects (for a detailed review see Antczak *et al.* 2016).

Toxoplasmosis is a representative of the urgent need for new antiprotozoal targets. In addition to the fact that *T. gondii* is estimated to infect 2–3 billion people worldwide (Welti *et al.* 2007), its treatment is complicated due to two main factors: (a) the parasite undergoes a complex life cycle with two predominant forms in the human host, namely, tachyzoites (proliferative form) and bradyzoites (encysted form, chronic toxoplasmosis); (b) bradyzoite burden is widespread but usually asymptomatic, although it has been associated with psychiatric disorders (Webster *et al.* 2013). However, in immunocompromised individuals encysted *T. gondii* transform into proliferative tachyzoite forms causing symptomatic disease, toxoplasmic encephalitis. As such *T. gondii* is an opportunistic parasite. Notably, all the above-mentioned drugs act only against the tachyzoite stage with no notable effect against encysted bradyzoites (Antczak *et al.* 2016). Recent data from our laboratory (Alqaisi *et al.* 2017) and others (Sonda *et al.* 2005) have shown that the Aureobasidin A and analogous depsipeptides, known to target yeast SL biosynthesis (Wuts *et al.* 2015), exhibit activity against bradyzoite *T. gondii.* This class of compounds may offer a potential treatment for chronic toxoplasmosis and, perhaps, some psychiatric disorders; although the mechanism of action is not via inhibition of parasite SL biosynthesis and is yet to be elucidated (Alqaisi *et al.* 2017).

#### Plasmodium falciparum

Falciparum malaria remains one of the ‘Big Three’, most prevalent and deadly infectious diseases across tropical and subtropical regions, with an estimated 154–289 million cases in 2010 (212 million cases in 2015), and 660 000 (429 000 in 2015) associated deaths; although the actual numbers might be even higher (Biamonte *et al.* 2013; WHO, 2016).

Similar to *T. gondii*, *Plasmodium* parasite undergoes a complex life cycle with different stages in different organs of the host, rendering treatment challenging: sporozoites and schizonts in the liver, and merozoites, trophozoites and gametocytes in the blood (Dechy-Cabaret & Benoit-Vical, 2012). Artemisinin-based combination therapies (ACTs) are the standard for treating malaria cases with typical partner drugs including lumefantrine and piperaquine, e.g. Coartem™ (Novartis) and Eurartesim™ (Sigma-Tau) (Biamonte *et al.* 2013). Other regimens include the use of parenteral artesunate (severe malaria) (Dondorp *et al.* 2010*a*), primaquine (liver and transmission, gametocyte, stages) (Dondorp, 2013), mefloquine and sulfadoxine/pyrimethamine in combination (effective as single dose antimalarial drug) (Biamonte *et al.* 2013) and atovaquone/proguanil, Malarone™ (GlaxoSmith Kline), as a prophylactic treatment.

However, although combination therapies have now been adopted, resistance against many existing antimalarials has been observed since the 1950s (Bishop, 1951; Hallinan, 1953; Sandosham *et al.* 1964) and remains a severe threat (Rieckmann & Cheng, 2002; Chinappi *et al.* 2010; Dondorp *et al.* 2010*b*; Newton *et al.* 2016; Parija, 2016; Menard & Dondorp, 2017; Zhou *et al.* 2017). This bleak view of the future of available anti-malarial chemotherapeutics makes it imperative to invest more efforts in identifying new potent chemotypes that will offer both efficacy and safety.

#### *Cryptosporidium* spp

Like *T. gondii*, *Cryptosporidium parvum* and *Cryptosporidium hominis* usually cause a self-limiting disease in healthy individuals but represent a manifest problem in immuno-compromised patients, particularly those with AIDS, where infection leads to acute and protracted life-threatening gastroenteritis (Chen *et al.* 2002). More recent data have led to a radical reassessment of the impact of cryptosporidiosis, with the number of *Cryptosporidium*-attributable diarrhoea episodes estimated at >7·5 million in children aged <24 months in sub-Saharan Africa and South Asia where infection is estimated to contribute to >250 000 infant deaths per year (Sow *et al.* 2016). Current treatment of cryptosporidiosis relies on a single FDA-approved drug, nitazoxanide, which has limited efficacy in those most at risk. More recently, the repurposing of antimalarials, e.g. quinolones and allopurinols, has been proposed (Gamo *et al.* 2010; Chellan *et al.* 2017).

The distinctive metabolic features of this parasite from other apicomplexan organisms, e.g. no plastid-derived apicoplast and the absence of the citrate cycle and cytochrome-based respiratory chain (Ryan & Hijjawi, 2015), confer several limitations for the identification of targets necessary for the development of anticryptosporidial drugs. However, the core metabolic pathways, e.g. energy metabolism and lipid synthesis are still present and exhibit high level of divergence from the mammalian host, thus presenting an opportunity to identify new drug targets that promise effective and selective treatment (Chellan *et al.* 2017).

### The biological significance of SLs

SLs are a class of lipids that are ubiquitous in eukaryotic cell membranes, particularly the plasma membrane, as well as in some prokaryotic organisms and viruses (Merrill & Sandhoff, 2002). Since their earliest characterization by Thudichum (1884), they have been a subject of controversy. Initially, they had been considered of structural importance only; however, over the last couple of decades, several reports have revealed their indispensability to a plethora of functions including, but not limited to, the formation of structural domains, polarized cellular trafficking, signal transduction, cell growth, differentiation and apoptosis (Huwiler *et al.* 2000; Ohanian & Ohanian, 2001; Cuvillier, 2002; Pettus *et al.* 2002; Buccoliero & Futerman, 2003).

**Fig. 1.**
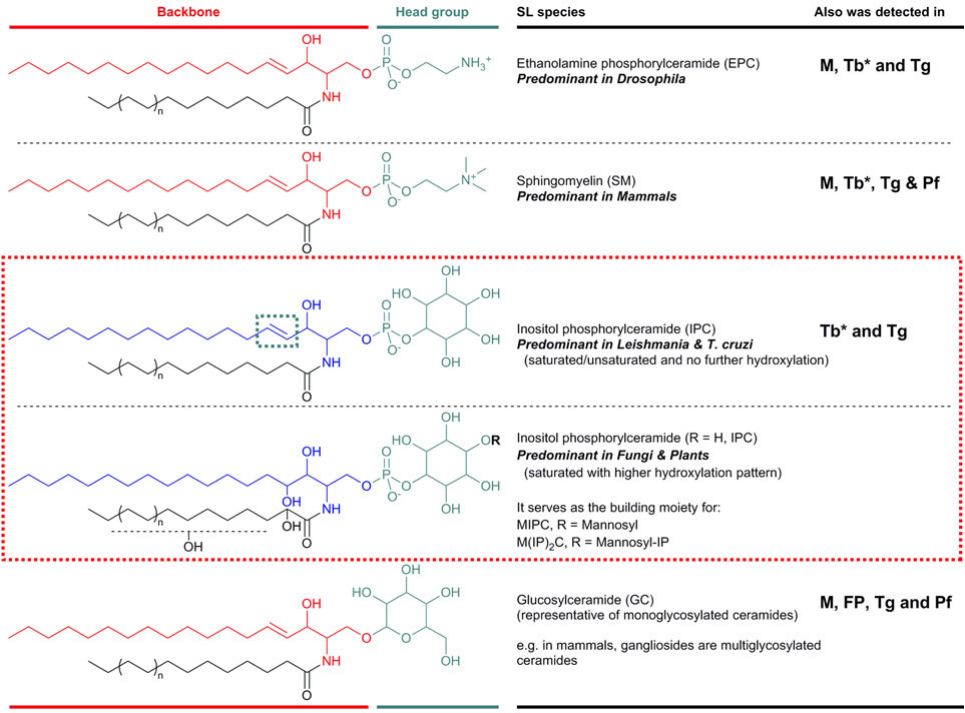
Illustration of the predominant species of complex sphingolipid in organisms from different evolutionary clades: EPC in Drosophila; SM in mammals; and IPC in Leishmania and *T. cruzi* (as representatives of protozoan parasites) and in fungi and plants. IPC is absent from Mammalian cells but essential for many pathogenic organisms (red box). Glycosylated sphingolipids are also ubiquitous across different species. Backbone chain length is commonly C18 derived from palmitoyl-CoA. Mammals M, Fungi and Plants FP, *Leishmania* spp. L, *Trypanosoma cruzi* Tc, *Trypanosoma brucei* Tb, *Toxoplasma gondii* Tg and *Plasmodium falciparum* Pf. *Denotes developmental regulation. EPC, ethanolamine phosphorylceramide; IPC, inositol phosphorylceramide; SM, sphingomyelin.

SLs consist structurally of a sphingoid base backbone, e.g. sphingosine (SPH) that can be *N*-acylated to form CER. To the latter, a variety of head groups: charged, neutral, phosphorylated and/or glycosylated can be attached to form complex SLs, e.g. sphingomyelin (SM), as the primary complex mammalian SL; and inositol phosphorylceramide (IPC) in fungi, plants and numerous protozoa (Fig. 1). These molecules have both polar and non-polar regions giving rise to their amphipathic character, which accounts for their tendency to aggregate into membranous structures, yet retaining the interfacial ability to interact with various partners, e.g. involvement of glycosphingolipids (GSLs) in cellular recognition complexes, cell adhesion and the regulation of cell growth (Gurr *et al.* 2002). Furthermore, the diversity of their chemical structures allows for distinctive roles within cellular metabolism, e.g. the signalling functions of SPH and CER *vs* sphingosine-1-phosphate (S1P) and ceramide-1-phosphate (C1P) (Merrill & Sandhoff, 2002; Metzler, 2003).

### SLs as indispensable structural components

The unique structural features of SLs (the free 3-hydroxy group, the amide functionality and the C4–C5 *trans* double bond) affect their biophysical properties rendering these molecules different from their glycerolipid counterparts, i.e. SM *vs* phosphatidylcholine (PC) (Boggs, 1980, 1987; Talbott *et al.*, 2000; Ramstedt & Slotte, 2002). Such interfacial differences give complex SLs, such as SM, the unique ability to form both *intra-* and *inter*molecular hydrogen bonds (Bruzik, 1988) that are fine-tuned by the *trans* double bond (Ramstedt & Slotte, 2002). This ability is reflected in the tendency of SLs to cluster rather than behave like typical ‘fluid’ membrane lipids. Naturally occurring SLs undergo the *L*_β_ (gel phase) to *L*_α_ (lamellar phase) transition near the physiological temperature of 37 °C, in contrast, this transition for naturally occurring glycerolipids is near or below 0 °C. Additionally, the long saturated alkyl chains of SLs allow them to pack tightly with sterols, stabilized by hydrogen bonding (Ramstedt & Slotte, 2002), to form laterally compact hydrophobic micro-domains commonly known as ‘lipid rafts’ (Futerman & Hannun, 2004). Similar results have been reported with the fungal/plant counterpart of SM, IPC, where it was shown that IPC was able to form sterol containing ordered domains in model systems (Björkbom *et al.* 2010). These membrane micro-domains can readily segregate from the more disordered and expanded domains of unsaturated acyl chains of glycerolipids (Merrill & Sandhoff, 2002). They have been proposed to function in a diverse array of processes from polarised trafficking of lipid modified proteins (Brown & London, 1998) and the stabilization of other types of biological structures such as lamellar bodies, to the assembly and activation of signal transduction complexes (Brown & London, 2000; Magee *et al.* 2002; Pierce, 2002; Vance & Vance, 2002; Hannun & Obeid, 2008). They have also been involved in the formation of detergent-insoluble gel-phase domains (Ramstedt & Slotte, 2002) via the extensive hydrogen-bonding network in the head groups of GSLs that have been implicated during the formation of ‘caveolae’ and surface recognition (Merrill & Sandhoff, 2002).

### SLs as indispensable signalling agents

SLs can also function as bioactive signalling molecules due to their biophysical properties, e.g. the low *pK*_*a*_ (7–8) of SPH allows it to remain partially uncharged at physiological pH retaining the ability to move across membranes (Merrill & Sandhoff, 2002). Likewise, CER, a neutral species, is able to freely flip flop across membranes (Hannun & Obeid, 2008). Many studies have produced evidence of such signalling functions, e.g. SPH exerts pleiotropic effects on protein kinases; CER mediates many cell-stress responses, including the regulation of apoptosis (Georgopapadakou, 2000); and S1P has crucial roles in cell survival, cell migration and inflammation (Hannun & Obeid, 2008)

### SL metabolism and the rationale for druggability

The indispensability of SLs for a myriad of cellular processes and functions, ranging from structural integrity to signalling events, makes it is unsurprising that the SL biosynthesis is highly conserved in all eukaryotes where it is, alongside its proposed regulators (Holthuis *et al.* 2006), an essential pathway (Heung *et al.* 2006; Sutterwala *et al.* 2007). This has lead the pathway to be considered vital for protozoan pathogenesis and, therefore, a drug target; e.g. SM synthase activity in *Plasmodium* (Heung *et al.* 2006). In order to characterise the druggability of protozoan SL biosynthesis, the mammalian pathway, as the most studied system, will be used as the reference model in the following discussions.

SL metabolism constitutes a highly complex network involving critical intersections with various other pathways, particularly glycerolipid biosynthesis (Holthuis & Menon, 2014). CER represents the corner stone for both biosynthesis and catabolism, modulating cell fate (Hannun & Obeid, 2008). Dysregulation of either SL biosynthesis or catabolism could result in cell death, e.g. of protozoan parasites (Yatsu, 1971; Brady, 1978; Chen *et al.* 1999; Merrill & Sandhoff, 2002), however here our focus will be on the former pathway.

**Fig. 2.**
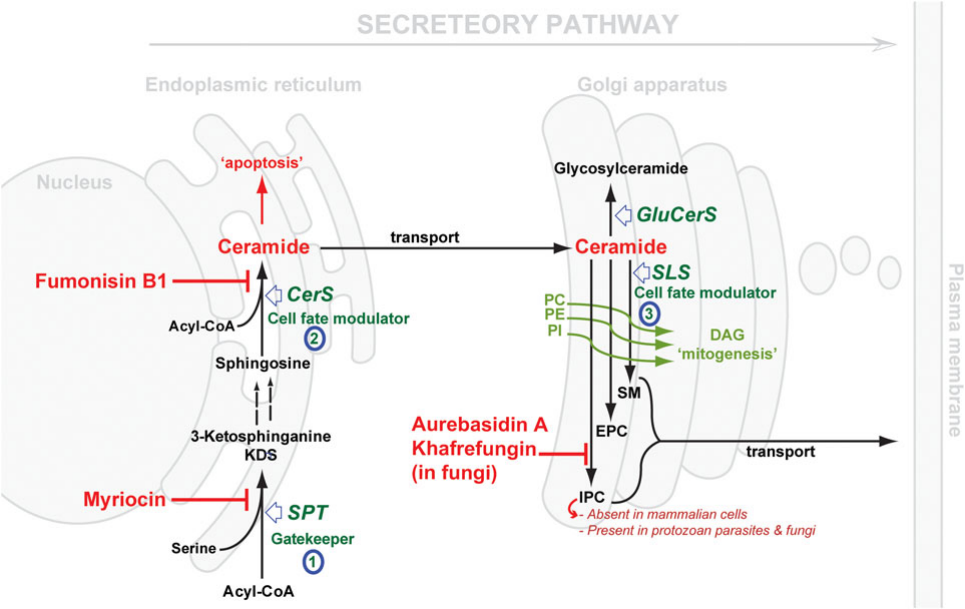
Schematic representation of *de novo* sphingolipid metabolism. Three key steps are highlighted: (1) SPT, evolutionary divergent in *T. gondii*; (2) CerS, fewer isoforms in protozoan parasite (c.f. 6 isoforms in mammals); SLS, while predominantly synthesising SM in mammals and to a lesser extent EPC, orthologues in protozoan parasites (*Leishmania* spp., *T. brucei*, *T. cruzi* and *T. gondii*) can synthesise IPC, an activity that is absent from mammalian cells and the target of the highly specific fungal inhibitors shown. The scheme also illustrates the differential cellular effects of ceramide *vs* DAG (diacylglycerol). Accumulation of ceramide elicits an apoptotic response while increasing concentrations of DAG promotes cell growth. CerS, ceramide synthase; GluCerS, glucosylceramide synthase; SLS, sphingolipid synthase; SPT, serine palmitoyltransferase; PC, phosphatidylcholine; PE, phosphatidylethanolamine; PI, phosphatidylinositol; SM, sphingomyelin; EPC, ethanolamine phosphorylceramide and IPC, inositol phosphorylceramide.

Considering the central position of CER, the druggability of SL metabolism revolves around dysregulation of ‘Ceramide Homeostasis’ (Young *et al.* 2012) which in turn leads to ripple effects perturbing the balance between the pro-apoptotic CER and the mitogenic diacylglycerol (DAG), consequently determining cell fate (Fig. 2) – a mechanism that has been associated with resistance to anti-cancer treatments (Ségui *et al.* 2006) and has been reported in protozoan parasites, e.g. Plasmodium (Pankova-Kholmyansky *et al.* 2003; Labaied *et al.* 2004). The characterisation of several key enzymes involved in SL *de novo* biosynthesis has revealed divergence between mammalian and protozoan species. Thus, attention has been given to the exploitation of the SL biosynthetic pathway (parasite and/or host) for new drug targets or regimens (Sugimoto *et al.* 2004; Zhang *et al.* 2005; Denny *et al.* 2006; Tanaka *et al.* 2007; Pruett *et al.* 2008; Mina *et al.* 2009; Tatematsu *et al.* 2011; Young *et al.* 2012).

## SL METABOLISM

### The key steps in de novo biosynthesis

SL *de novo* biosynthesis can be simplified into three key steps: a gate-keeper and two cell fate modulator steps. The former comprises the up-stream rate-limiting step of the condensation of acyl-CoA and L-serine, in the endoplasmic reticulum (ER) via serine palmitoyltransferase (SPT), to produce dihydrosphingosine. The latter comprises first the formation of CER in the ER by the action of ceramide synthase (CerS), and then the formation of complex SLs in the Golgi. These products vary depending on the species, and are formed under the catalysis of what could be generically termed SL synthases: SM synthase in mammals and IPC synthase in fungi, plants and protozoa. It is worth mentioning that another Golgi localized metabolic pathway results in the formation of glycosylated CER species, and also contributes to the regulation CER levels (Holthuis & Menon, 2014) (Fig. 2).

### Protozoan parasites *vs* host: differences & opportunities

The cross-species differences encountered in the first, SPT-catalysed, step are mostly minor in terms of the chemical structure of the product; mainly due to the chain length of the acyl-CoA utilised in the reaction, e.g. myristoyl-CoA (in *Leishmania* spp. amongst other odd sphingoid base lengths (Hsu *et al.* 2007)) and palmitoyl-CoA, with the latter more predominant across the Eukaryota (in mammals, *Plasmodium* and *T. brucei*) (Richmond *et al.* 2010; Botté *et al.* 2013). Further differences may be apparent with respect to the catalysing enzyme, SPT (*vide infra*). However, clear divergence is observed in the second and the third steps, both of which represent a cell-fate modulator process. CerSs exhibit differential preferences for the chain length of the acyl-CoA substrate (Park *et al.* 2014) and its hydroxylation pattern (Layre & Moody, 2013), with 6 isoforms present in humans suggesting a different role for each CER species produced (Levy & Futerman, 2010; Figueiredo *et al.* 2012). To-date, one or, maximum, two genes encoding CerS function have been identified in protozoan parasite species (Koeller & Heise, 2011). However, most interesting is the variation in the complex SL formed in the Golgi, reflecting significant differences in the active site of the SL synthases catalysing the transfer reaction. The divergence of the protozoal complex SL synthases, and the synthetic products, with respect to the mammalian host, may provide opportunities to design selective inhibitors. Previously, this step has been validated as a promising drug target in fungi using aureobasidin A (AbA) (Fig. 2) (Denny *et al.* 2006).

### Serine palmitoyl transferase (SPT)

SPTs are members of the pyridoxal 5′-phosphate (PLP)-dependent (Sandmeier *et al.* 1994) α-oxoamine synthase family and share a conserved motif (T[FL][GTS]**K**[SAG][FLV]G) around the PLP-binding lysine (in bold) (Young *et al.* 2012). SPT catalyses the first rate-limiting step in the *de novo* biosynthesis of SLs (Weiss & Stoffel, 1997; Hojjati *et al.* 2005) (Fig. 2), a reaction involving the decarboxylative Claisen-like condensation of serine and an acyl-CoA (Lowther *et al.* 2012), to yield the sphingoid base backbone, 3-ketodihydrosphingosine (3-KDS) (Hanada, 2003; Raman *et al.* 2009; Lowther *et al.* 2012). Therefore, SPT represents the ‘Gatekeeper’ of the SL biosynthetic pathway.

All eukaryotic SPTs studied to date are ER-resident and membrane bound with a heterodimeric protein core consisting of two subunits sharing ~20% identity: LCB1 and LCB2, ~53 and ~63 kDa respectively (Hanada, 2003; Denny *et al.* 2004; Han *et al.* 2004; Chen *et al.* 2006). The latter contains the canonical PLP cofactor binding site while the former has been suggested to be important for complex stability (Lowther *et al.* 2012). In contrast, the orthologous SPT from sphingomonad bacteria is a soluble 45 kDa homodimer (Ikushiro *et al.* 2001). SPT activity in apicomplexan parasites has been detected and was proposed as a potential drug target (Gerold & Schwarz, 2001; Bisanz *et al.* 2006; Coppens, 2013), however the enzyme(s) responsible have yet to be further characterized (Mina *et al.* 2017). In contrast, kinetoplastid parasites have been shown to possess a heterodimeric SPT similar to the mammalian orthologue (Denny *et al.* 2004). Inhibiting SPT activity (e.g. using myriocin, Fig. 2) results in various effects in different species. Mammalian cells exhibited a loss of viability, with a partial loss of SPT function resulting in a rare SL metabolic disease, Hereditary Sensory Neuropathy type I (HSN1) (Hanada, 2003). In contrast, *Saccharomyces cerevisiae* were found to be relatively tolerant (Nagiec *et al.* 1994), and *Leishmania major* lacking LCB2 were viable but unable to differentiate into infective metacyclic forms (Zhang *et al.* 2003). However, *T. brucei* procyclic forms in which SPT expression was reduced were non-viable (Fridberg *et al.* 2008).

The SPT catalysed reaction product, 3KDS, is subsequently reduced by 3-ketosphinganine reductase to form sphinganine (dihydrosphingosine). Subsequent minor metabolic differences are encountered across different species; mainly concerning the order of the hydroxylation (in fungi and higher plants) and acylation to produce CERs (Sugimoto *et al.* 2004).

### Ceramide synthase

In all eukaryotic systems studied to date, CerSs are ER-resident integral membrane proteins catalysing the *N*-acetylation of dihydrosphingosine to produce dihydroceramide, which is then oxidized to form CER, the simplest SL species and a key bioactive molecule in numerous cellular pathways (Lahari & Futerman, 2007).

Mammalian CerSs are orthologues of longevity-assurance genes, LAG1p and LAC1p identified in yeast (Guillas *et al.* 2001). The eukaryotes studied to date have been found to encode at least two CerSs, with humans expressing six – each generating CER with a defined acyl chain length (C18 to C26) (Pewzner-Jung *et al.* 2006; Levy & Futerman, 2010). Whilst little is known regarding structure-function relationships or regulation of CerS,, the ubiquitous Lag1 motif has been shown to be important for functionality (Spassieva *et al.* 2006), likely forming part of the active site.

Experimental evidence (from our laboratory and others) has previously indicated the presence of CerS activity in *Leishmania* spp (Zhang *et al.*, 2003; Denny *et al.*, 2004, 2006) and in *T. cruzi* (De Lederkremer *et al.* 2011). More recently LAG1 orthologues have been identified and functionally and molecularly characterized in the latter parasite (Figueiredo *et al.* 2012). Other results indirectly suggest the presence of such activity in *T. brucei* (Patnaik *et al.* 1993; Richmond *et al.* 2010; Smith & Bütikofer, 2010). Similarly, CerS activity in the Apicomplexa has been inferred (Welti *et al.* 2007; Zhang *et al.* 2010; Pratt *et al.* 2013), but remains unexplored.

Once formed in the ER, CER is transported, by CER transfer protein CERT in mammals (Kumagai *et al.* 2005; Kudo *et al.* 2010; Rao *et al.* 2014), to the Golgi apparatus where the synthesis of complex SLs occurs (Ohanian & Ohanian, 2001; Bromley *et al.* 2003; Bartke & Hannun, 2009; Pata *et al.* 2010). ER CER concentration is kept under tight control as accumulation of CER here has been shown to result in induction of the mitochondrial apoptotic pathway (Vacaru *et al.* 2009; Tafesse *et al.* 2014) via an unknown mechanism (Bockelmann *et al.* 2015).

### Sphingolipid synthase

In the Golgi, CER can be phosphorylated by CER kinase (Rovina *et al.* 2009), glycosylated by glucosyl or galactosyl CerS (Raas-Rothschild *et al.* 2004), or acquire a variety of neutral or charged head groups under the catalysis of what could be called generically SLSs, to form various complex phosphosphingolipids. Phylogenetic analyses have identified at least 4 clades of SLS (Huitema *et al.* 2004; Denny *et al.* 2006).

In mammals CER is a substrate for the SLS, SM synthase, to produce SM (Huitema *et al.* 2004). Whilst in fungi and higher plants phytoceramide is utilized by a different SLS, IPC synthase, to produce IPC as the principal phosphosphingolipid (Nagiec *et al.* 1997; Wang *et al.* 2008). This landscape is significantly divergent when it comes to protozoa.

In the kinetoplastid *Leishmania* spp. and *T. cruzi*, CER acquires a phosphorylinositol head group from phosphatidylinositol (PI) to produce IPC via IPC synthase (Zhang *et al.* 2005; Denny *et al.* 2006; Mina *et al.* 2010), although there are some reports of SM in *T. cruzi* (Quiñones *et al.* 2004) (Fig. 2). Whilst *Leishmania* encodes a single copy IPC synthase, *T. cruzi* has two highly related copies (Denny *et al.* 2006). Further divergence, and possible redundancy, is encountered in *T. Brucei*, which harbours 4 genes that encode SLSs (Denny *et al.* 2006; Sutterwala *et al.* 2008). This enzyme portfolio results in a diverse profile of the complex SL species (SM, IPC and ethanolamine phosophorylceramide [EPC]) which are developmentally regulated during the life cycle of the parasite (Sutterwala *et al.* 2008).

In apicomplexan parasites, previous reports have indicated the presence of glycosyl-ceramide and SM in *P. falciparum* and *T. gondii*, as summarized in Zhang *et al* (2010). However, other findings reported the presence of EPC in *T. gondii* (Welti *et al.* 2007) and, more recently, IPC (Pratt *et al.* 2013). The latter study also characterized *T. gondii* SLS as demonstrating IPC synthase activity *in vitro* (Pratt *et al.* 2013).

The divergence of SLS function, with respect to the host, seen in both kinetoplastid and apicomplexan protozoan parasites in intriguing and, perhaps, indicated them as a tractable drug target. In support of this hypothesis, ceramide-analogues with anti*-Plasmodium* activity have already been identified (Labaied *et al.* 2004).

In general, SLSs are Golgi-resident transmembrane proteins, presumed to have 6 transmembrane domains with the active site facing the Golgi lumen (Holthuis *et al.* 2006; Sutterwala *et al.* 2008). Those orthologues identified in kinetoplastids demonstrated two conserved regions (CGD*X*_3_SG**H**T & **H**YT*X***D**V*X*_3_Y*X*_6_F*X*_2_YH) with respect to the animal SM synthases (Huitema *et al.* 2004; Denny *et al.* 2006). These regions contain the so-called the catalytic triad (two Histidines and one Aspartate residues) that mediates a nucleophilic attack on lipid phosphate ester during the transferase/hydrolase activity (Mina *et al.* 2010). Apicomplexan orthologues form a separate evolutionary clade, yet retain the catalytic triad (Denny *et al.* 2006; Pratt *et al.* 2013), as does the fungal orthologue AUR1p (Heidler & Radding, 2000). Further evidence for the essentiality of these residues was provided when mutation of the active histidine of the triad was shown to deactivate fungal IPC synthase and mammalian SM synthase-related activity (Levine *et al.* 2000; Vacaru *et al.* 2009). Furthermore, recently it has been shown that substrate selectivity, and so the diversity of SLS activity, may depend on key residues close to the transferase active residues or on a luminal loop of the protein (Sevova *et al.* 2010; Kol *et al.* 2017).

In the Eukaryota SLS's occupy a central position at the intersection of glycerolipids (PI/PC/PE and DAG) and SLs ([phyto]ceramide and IPC/SM/EPC). Accordingly, these enzymes act as regulators of a delicate balance between pro-apoptotic CER and pro-mitogenic DAG (Holthuis *et al.* 2006).

The most significant previous example of SL biosynthesis inhibition as a drug target was reported in fungi. Aureobasidin A (AbA), a depsipeptide, was first reported by Ikai *et al.* (1991) and soon after its antifungal properties were highlighted (Takesako *et al.* 1993). The target gene was further characterized (Hashidaokado *et al.* 1995) revealing its identity to be the IPC synthase (AUR1p). AbA is a specific and potent (low nanomolar) inhibitor of the fungal IPC synthase. This ushered in a new era in the search for anti-fungal chemotherapeutics, positioning IPC synthase as a promising, broad spectrum, anti-fungal drug target (Sugimoto *et al.* 2004). Other specific inhibitors were later added to the arsenal of fungal IPC synthase inhibitors: khafrefungin (Mandala *et al.* 1997), rustmicin (Harris *et al.* 1998; Mandala *et al.* 1998) and others (Ohnuki *et al.* 2009). Unfortunately, further development of these inhibitors stalled, either due to physical properties, e.g. aureobasidin A is very sparingly soluble in water (Georgopapadakou, 2000; Sugimoto *et al.* 2004), or because their highly complex chemical structures rendered chemical synthesis challenging, with the few synthetic efforts reported resulting in compounds with either reduced or no activity (Sugimoto *et al.* 2004; Aeed *et al.* 2009). However, recent works have highlighted that semi-synthetic strategies may overcome these barriers (Wuts *et al.* 2015).

Perhaps reflecting the evolutionary divergence of these enzymes, the protozoan IPC synthase orthologues, from *Leishmania major* and *T. gondii* are not susceptible to AbA inhibition (Denny *et al.* 2006; Pratt *et al.* 2013). Some studies have reported the inhibitory effects of AbA and analogues against *T. gondii* in culture (Sonda *et al.* 2005; Alqaisi *et al.* 2017), however this is not associated with inhibition of SL biosynthesis. Despite this, the protozoan SLS's remain tractable drug targets with no functional equivalent in mammalian cells. Surprisingly, at least one SLS isoform from *T. brucei* was acutely sensitive to AbA treatment (Mina *et al.* 2009), although these findings stirred some controversy due, in part, to the redundancy of *T. brucei* SLSs (4 isoforms) compared with the single copy found, for example, in *L. major* and *T. gondii* (Sutterwala *et al.* 2008).

## THE ENIGMATIC NATURE OF SL DRUGGABILITY

### Difficulties in pinpointing SL functionality

Investigation and deciphering of the functions of each specific SL species remains challenging. This is due to the complexity in SL metabolic interconnections, their varied biophysical properties (neutral or charged), chain length variation, the hydrophobic nature of the involved enzymes and the presence of multiple pathways that can operate in parallel (Hannun & Obeid, 2008). The interaction with other cellular metabolic pathways (e.g. glycerolipid metabolism) introduces another layer of complexity.

Overall, the signalling effect/role of an individual SL could be defined on spatial-temporal basis with at least five parameters: (a) subcellular localisation, (b) regulation (c) chain length specificity, (d) kinetics of trafficking and (e) mechanism of action. For example, phosphorylation of 1–3% cytosolic SPH may double the levels of S1P that acts on G protein-coupled receptor (GPCR) to elicit a specific response in a particular cellular locality for certain period of time (Hannun & Obeid, 2008). Such signalling events can be described as a function of cytosolic S1P that is regulated by S1P Kinase, with the signal caused through the interaction of S1P with a GPCR. The elucidation of such complex systems remains challenging and a comprehensive discussion of the issue is beyond the scope of this review. However, an additional layer of significant complexity in terms of the pathogenic protozoa arises when considering the SL signalling network in the case of obligate intracellular parasites, where host SL biosynthesis, and its interaction with parasite *de novo* synthesis, must be taken into account.

### Parasite–host SL interplay

The intimate parasite–host interaction in terms of SL metabolism has been well documented; *L. major* pathogenic amastigotes isolated from mammalian hosts showed normal IPC levels (Zhang *et al.* 2005) despite lacking LCB2, a functional SPT and the ability to synthesis CER *de novo*. Alterations in host, macrophage, cell SL biosynthesis upon infection may compensate for this deficiency (Ghosh *et al.*, 2001, 2002). These studies suggest a complex and multifaceted interplay between host and parasite SL metabolism comprising nutritional factors and signalling pathways that could modulate parasite survival and/or host defence (Zhang *et al.* 2010). Similar observations have been reported in the apicomplexan parasites (Romano *et al.* 2013). This highlights the striking potential of host and parasite SL modulation as an anti-protozoal target, as is similarly proposed for pathogenic fungi (Zhang *et al.* 2010; Ramakrishnan *et al.* 2013).

## PERSPECTIVE

Classically dissecting the role and locale of critical enzymatic steps in SL biosynthesis and assessing the effect on the parasite fitness and virulence could turn into an overwhelmingly challenging task aggravated by: the complexity of the metabolic pathway itself; the ability of the parasite to salvage (Coppens, 2013), hijack and remodel host SL; and developmental regulation during the parasitic life cycle, which adds another layer of intricacy rendering the deconvolution of any observed effects difficult to interpret. Fortunately, many of those problems can be now overcome with advances in technology. High resolution localization studies in protozoan parasites can benefit greatly from new microscopic techniques such as Airy-scan (Huff, 2015), super-resolution microscopy (Florentino *et al.* 2014) and upcoming technologies, e.g. phase-modulation nanoscopy (Pal, 2015; Ward & Pal, 2017), which can elucidate spatial arrangement of proteins of interest within the parasite to reveal potential interaction partners and shed light on mechanistic features. Similarly, new advances in chemical probes, and SL analogues in particular, such as bifunctional lipid technology (Haberkant & Holthuis, 2014) coupled with high throughput proteomic (Ramaprasad *et al.* 2015), could identify different interaction partners that would help map the biosynthetic pathway and its critical interactions. The effects of these probes on the parasite (and host) cell can now be comprehensively evaluated by monitoring the transcriptome, proteome, metabolomics (Watson, 2010) and lipidome (Marechal *et al.* 2011). Such studies could reveal multiple windows of opportunity to exploit as potential drug targets. The targets identified in this way can now be rapidly genetically validated in the parasitic protozoa by applying modern gene editing technologies, such as CRISPR/Cas9 (Sugi *et al.* 2016). Compared with the classical methodologies, this tool enables fast and efficient application for single gene (Serpeloni *et al.* 2016), and systematic genome-wide knockout generation (Sidik *et al.* 2016). Additionally, the development of novel orthogonal approach for conditional knockout strategies, e.g. tetracycline-induced gene disruption Tet-system (Meissner *et al.* 2002), rapamycin-induced Cre recombinase-assisted gene excision (Andenmatten *et al.* 2013; Collins *et al.* 2013; Jimenez-Ruiz *et al.* 2014), has allowed testing of essential gene functionality, in *Leishmania* spp. (Duncan *et al.* 2016) and *T. gondii* (Pieperhoff *et al.* 2015).

Aside from the increase ability to robustly validate targets such as SL biosynthesis, global collaboration between academia and pharmaceutical partners is expediting the process of drug discovery of new anti-protozoal drugs. For example, within the sphere of targeting SL biosynthesis in the protozoa, we have managed several projects with industrial partners, MRCT and Tres Cantos Open Lab Foundation (https://www.openlabfoundation.org, an initiative of GlaxoSmithKline), in the pursuit of identifying new compound scaffolds active against the *Leishmania* spp IPC synthase utilising yeast (Norcliffe *et al.* 2014) as a vehicle for drug discovery (Denny & Steel, 2015). The generated results and techniques could readily be translated to other disease targets. Other global initiatives include Open Innovation Drug Discovery, Eli Lilly, which is focused on cancer, cardiovascular disease, endocrine disorders, neuroscience and tuberculosis. The Centers for Therapeutic Innovation, facilitates Pfizer and academic researchers to work together in order to develop new biologics programs and WIPO Re:Search, provide participant researchers with access to patents and expertise related to drug discovery for 19 NTDs, malaria and tuberculosis (Sheridan, 2011).

Finally, SL biosynthesis represents a gold mine for new drug targets alongside at least two axes, *de novo* synthesis and salvage and remodelling. On one hand, the protozoan *de novo* SL biosynthetic pathway comprises three key steps, and considering their divergence compared with the mammalian host, identifying specific inhibitors for those could open an opportunity for anti-protozoal drugs with synergistic effects and lower incidences of resistance. On the other hand, the nature of obligate intracellular parasites dictates that further efforts should be directed towards the catabolic/salvage pathway where parasite–host dependencies could be exploited in order to identify additional key steps, or host enzymes, where inhibitors would exert further synergism with the *de novo* inhibitors.

To summarize, the landscape of anti-protozoan drug discovery requires immediate attention: with the re-evaluation of knowledge gained, the application of recent technologies; and the support of coordinated global discovery efforts. The multifaceted effects of SLs as a dynamic matrix of interaction (spatial and temporal) and function makes SL biosynthesis highly alluring for drug intervention, after all, everybody needs SLs, right?

## References

[ref1] Aeed P. A., Young C. L., Nagiec M. M. and Elhammer A. P. (2009). Inhibition of inositol phosphorylceramide synthase by the cyclic peptide aureobasidin A. Antimicrobial Agents and Chemotherapy 53, 496–504. 19047657 10.1128/AAC.00633-08 PMC2630602

[ref2] Alqaisi A. Q. I., Mbekeani A. J., Llorens M. B., Elhammer A. P. and Denny P. W. (2017). The antifungal Aureobasidin A and an analogue are active against the protozoan parasite *Toxoplasma gondii* but do not inhibit sphingolipid biosynthesis. Parasitology, 1–8. doi: 10.1017/S0031182017000506. PMC5964465 28486997

[ref3] Andenmatten N., Egarter S., Jackson A. J., Jullien N., Herman J. P. and Meissner M. (2013). Conditional genome engineering in *Toxoplasma gondii* uncovers alternative invasion mechanisms. Nature Methods 10, 125–127. 23263690 10.1038/nmeth.2301 PMC3605914

[ref4] Andrews K. T., Fisher G. and Skinner-Adams T. S. (2014). Drug repurposing and human parasitic protozoan diseases. International Journal for Parasitology: Drugs and Drug Resistance 4, 95–111. 25057459 10.1016/j.ijpddr.2014.02.002 PMC4095053

[ref5] Antczak M., Dzitko K. and Długońska H. (2016). Human toxoplasmosis-searching for novel chemotherapeutics. Biomedicine & Pharmacotherapy 82, 677–684. 27470411 10.1016/j.biopha.2016.05.041

[ref6] Babokhov P., Sanyaolu A. O., Oyibo W. A., Fagbenro-Beyioku A. F. and Iriemenam N. C. (2013). A current analysis of chemotherapy strategies for the treatment of human African trypanosomiasis. Pathogens and Global Health 107, 242–252. 23916333 10.1179/2047773213Y.0000000105 PMC4001453

[ref7] Baron E. J. (1996). Classification In Medical Microbiology, 4th edn. (ed. Baron S.). University of Texas Medical Branch at Galveston, Galveston, TX, USA. 21413252

[ref8] Bartke N. and Hannun Y. A. (2009). Bioactive sphingolipids: metabolism and function. Journal of Lipid Research 50, S91–S96 19017611 10.1194/jlr.R800080-JLR200 PMC2674734

[ref9] Bermudez J., Davies C., Simonazzi A., Pablo Real J. and Palma S. (2016). Current drug therapy and pharmaceutical challenges for Chagas disease. Acta Tropica 156, 1–16. 26747009 10.1016/j.actatropica.2015.12.017

[ref10] Biamonte M. A., Wanner J. and Le Roch K. G. (2013). Recent advances in malaria drug discovery. Bioorganic & Medicinal Chemistry Letters 23, 2829–2843. 23587422 10.1016/j.bmcl.2013.03.067 PMC3762334

[ref11] Bisanz C., Bastien O., Grando D., Jouhet J., Marechal E. and Cesbron-Delauw M. F. (2006). *Toxoplasma gondii* acyl-lipid metabolism: de novo synthesis from apicoplast-generated fatty acids versus scavenging of host cell precursors. Biochemical Journal 394, 197–205. 16246004 10.1042/BJ20050609 PMC1386017

[ref12] Bishop A. (1951). Drug-resistance in malaria. British Medical Bulletin 8, 47–50. 14944814 10.1093/oxfordjournals.bmb.a074053

[ref13] Björkbom A., Ohvo-Rekilä H., Kankaanpää P., Nyholm T. K. M., Westerlund B. and Slotte J. P. (2010). Characterization of membrane properties of inositol phosphorylceramide. Biochimica et Biophysica Acta *(*BBA*) –* Biomembranes 1798, 453–460 19913494 10.1016/j.bbamem.2009.11.003

[ref14] Black S. J. and Mansfield J. M. (2016). Prospects for vaccination against pathogenic African trypanosomes. Parasite Immunology 38, 735–743. 27636100 10.1111/pim.12387

[ref15] Bockelmann S., Mina J., Jain A., Ehring K., Korneev S. and Holthuis J. C. M. (2015). Molecular dissection of ceramide-induced apoptosis using bifunctional lipid analogs. Febs Journal 282, 399–399.

[ref16] Boggs J. M. (1980). Intermolecular hydrogen-bonding between lipids – influence on organization and function of lipids in membranes. Canadian Journal of Biochemistry 58, 755–770. 7006755 10.1139/o80-107

[ref17] Boggs J. M. (1987). Lipid intermolecular hydrogen-bonding – influence on structural organization and membrane-function. Biochimica et Biophysica Acta 906, 353–404. 3307919 10.1016/0304-4157(87)90017-7

[ref18] Botté C. Y., Yamaryo-Botté Y., Rupasinghe T. W. T., Mullin K. A., MacRae J. I., Spurck T. P., Kalanon M., Shears M. J., Coppel R. L., Crellin P. K., Maréchal E., McConville M. J. and McFadden G. I. (2013). Atypical lipid composition in the purified relict plastid (apicoplast) of malaria parasites. Proceedings of the National Academy of Sciences of the United States of America 110, 7506–7511. 23589867 10.1073/pnas.1301251110 PMC3645554

[ref19] Brady R. O. (1978). Sphingolipidoses. Annual Review of Biochemistry 47, 687–713. 10.1146/annurev.bi.47.070178.003351 98102

[ref20] Bray P. G., Barrett M. P., Ward S. A. and de Koning H. P. (2003). Pentamidine uptake and resistance in pathogenic protozoa: past, present and future. Trends in Parasitology 19, 232–239. 12763430 10.1016/s1471-4922(03)00069-2

[ref21] Bromley P. E., Li Y. N. O., Murphy S. M., Sumner C. M. and Lynch D. V. (2003). Complex sphingolipid synthesis in plants: characterization of inositolphosphorylceramide synthase activity in bean microsomes. Archives of Biochemistry and Biophysics 417, 219–226. 12941304 10.1016/s0003-9861(03)00339-4

[ref22] Brown D. A. and London E. (1998). Functions of lipid rafts in biological membranes. Annual Review of Cell and Developmental Biology 14, 111–136. 10.1146/annurev.cellbio.14.1.111 9891780

[ref23] Brown D. A. and London E. (2000). Structure and function of sphingolipid- and cholesterol-rich membrane rafts. Journal of Biological Chemistry 275, 17221–17224. 10770957 10.1074/jbc.R000005200

[ref24] Bruzik K. S. (1988). Conformation of the polar headgroup of sphingomyelin and its analogues. Biochimica et Biophysica Acta (BBA) – Biomembranes 939, 315–326. 3355820 10.1016/0005-2736(88)90076-4

[ref25] Buccoliero R. and Futerman A. H. (2003). The roles of ceramide and complex sphingolipids in neuronal cell function. Pharmacological Research 47, 409–419. 12676515 10.1016/s1043-6618(03)00049-5

[ref26] Buckner F. S., Waters N. C. and Avery V. M. (2012). Recent highlights in anti-protozoan drug development and resistance research. International Journal for Parasitology: Drugs and Drug Resistance 2, 230–235. 24533285 10.1016/j.ijpddr.2012.05.002 PMC3862445

[ref27] Carabarin-Lima A., González-Vázquez M. C., Rodríguez-Morales O., Baylón-Pacheco L., Rosales-Encina J. L., Reyes-López P. A. and Arce-Fonseca M. (2013). Chagas disease (American trypanosomiasis) in Mexico: an update. Acta Tropica 127, 126–135. 23643518 10.1016/j.actatropica.2013.04.007

[ref28] CDC (2017). About Parasites. Centers for Disease Control and Prevention, Atlanta, GA, USA https://www.cdc.gov/parasites/about.html

[ref29] Center for Food Security and Public Health, C. o. V. M., Iowa State University, Ames, Iowa 50011 (2004). Leishmaniasis (cutaneous and visceral). Center for Food Security and Public Health, College of Veterinary Medicine, Iowa State University, Ames, Iowa 50011.

[ref30] Chappuis F., Sundar S., Hailu A., Ghalib H., Rijal S., Peeling R. W., Alvar J. and Boelaert M. (2007). Visceral leishmaniasis: what are the needs for diagnosis, treatment and control? Nature Reviews Microbiology, 5, 873–882. 17938629 10.1038/nrmicro1748

[ref31] Chellan P., Sadler P. J. and Land K. M. (2017). Recent developments in drug discovery against the protozoal parasites Cryptosporidium and Toxoplasma. Bioorganic & Medicinal Chemistry Letters 27, 1491–1501. 28242275 10.1016/j.bmcl.2017.01.046

[ref32] Chen C.-S., Patterson M. C., Wheatley C. L., O'Brien J. F. and Pagano R. E. (1999). Broad screening test for sphingolipid-storage diseases. Lancet 354, 901–905. 10489949 10.1016/S0140-6736(98)10034-X

[ref33] Chen M., Han G., Dietrich C. R., Dunn T. M. and Cahoon E. B. (2006). The essential nature of sphingolipids in plants as revealed by the functional identification and characterization of the Arabidopsis LCB1 subunit of serine palmitoyltransferase. Plant Cell 18, 3576–3593. 17194770 10.1105/tpc.105.040774 PMC1785403

[ref34] Chen X.-M., Keithly J. S., Paya C. V. and LaRusso N. F. (2002). Cryptosporidiosis. New England Journal of Medicine 346, 1723–1731. 12037153 10.1056/NEJMra013170

[ref35] Chinappi M., Via A., Marcatili P. and Tramontano A. (2010). On the mechanism of chloroquine resistance in *Plasmodium falciparum*. Plos ONE 5, e14064. 21124966 10.1371/journal.pone.0014064 PMC2988812

[ref36] Collins C. R., Das S., Wong E. H., Andenmatten N., Stallmach R., Hackett F., Herman J. P., Muller S., Meissner M. and Blackman M. J. (2013). Robust inducible Cre recombinase activity in the human malaria parasite *Plasmodium falciparum* enables efficient gene deletion within a single asexual erythrocytic growth cycle. Molecular Microbiology 88, 687–701. 23489321 10.1111/mmi.12206 PMC3708112

[ref37] Colwell D. D., Dantas-Torres F. and Otranto D. (2011). Vector-borne parasitic zoonoses: emerging scenarios and new perspectives. Veterinary Parasitology 182, 14–21. 21852040 10.1016/j.vetpar.2011.07.012

[ref38] Coppens I. (2013). Targeting lipid biosynthesis and salvage in apicomplexan parasites for improved chemotherapies. Nature Reviews Microbiology 11, 823–835. 24162026 10.1038/nrmicro3139

[ref39] Croft S. L. and Coombs G. H. (2003). Leishmaniasis – current chemotherapy and recent advances in the search for novel drugs. Trends in Parasitology 19, 502–508. 14580961 10.1016/j.pt.2003.09.008

[ref40] Croft S. L., Sundar S. and Fairlamb A. H. (2006). Drug resistance in leishmaniasis. Clinical Microbiology Reviews 19, 111–126. 16418526 10.1128/CMR.19.1.111-126.2006 PMC1360270

[ref41] Cuvillier O. (2002). Sphingosine in apoptosis signaling. Biochimica et Biophysica Acta *(*BBA*) –* Molecular and Cell Biology of Lipids 1585, 153–162. 12531549 10.1016/s1388-1981(02)00336-0

[ref42] Dechy-Cabaret O. and Benoit-Vical F. (2012). Effects of antimalarial molecules on the gametocyte stage of *Plasmodium falciparum*: the debate. Journal of Medicinal Chemistry 55, 10328–10344. 23075290 10.1021/jm3005898

[ref43] De Lederkremer R. M., Agusti R. and Docampo R. (2011). Inositolphosphoceramide metabolism in *Trypanosoma cruzi* as compared to other Trypanosomatids. Journal of Eukaryotic Microbiology 58, 79–87. 21332877 10.1111/j.1550-7408.2011.00533.x PMC3444516

[ref44] de Oliveira C. I., Nascimento I. P., Barral A., Soto M. and Barral-Netto M. (2009). Challenges and perspectives in vaccination against leishmaniasis. Parasitology International 58, 319–324. 19698801 10.1016/j.parint.2009.07.013

[ref45] Denny P. W. and Steel P. G. (2015). Yeast as a potential vehicle for neglected tropical disease drug discovery. Journal of Biomolecular Screening 20, 56–63. 25121554 10.1177/1087057114546552

[ref46] Denny P. W., Goulding D., Ferguson M. A. and Smith D. F. (2004). Sphingolipid-free Leishmania are defective in membrane trafficking, differentiation and infectivity. Molecular Microbiology 52, 313–327. 15066023 10.1111/j.1365-2958.2003.03975.x

[ref47] Denny P. W., Shams-Eldin H., Price H. P., Smith D. F. and Schwarz R. T. (2006). The protozoan inositol phosphorylceramide synthase: a novel drug target that defines a new class of sphingolipid synthase. Journal of Biological Chemistry 281, 28200–28209. 16861742 10.1074/jbc.M600796200 PMC1817671

[ref48] Dondorp A. M. (2013). Editorial commentary: single-dose primaquine as gametocytocidal treatment in patients with uncomplicated falciparum malaria. Clinical Infectious Diseases, 56, 694–696. 23175557 10.1093/cid/cis962 PMC3563393

[ref49] Dondorp A. M., Fanello C. I., Hendriksen I. C. E., Gomes E., Seni A., Chhaganlal K. D., Bojang K., Olaosebikan R., Anunobi N., Maitland K., Kivaya E., Agbenyega T., Nguah S. B., Evans J., Gesase S., Kahabuka C., Mtove G., Nadjm B., Deen J., Mwanga-Amumpaire J., Nansumba M., Karema C., Umulisa N., Uwimana A., Mokuolu O. A., Adedoyin O. T., Johnson W. B. R., Tshefu A. K., Onyamboko M. A., Sakulthaew T., Ngum W. P., Silamut K., Stepniewska K., Woodrow C. J., Bethell D., Wills B., Oneko M., Peto T. E., von Seidlein L., Day N. P. J. and White N. J. (2010 *a*). Artesunate versus quinine in the treatment of severe falciparum malaria in African children (AQUAMAT): an open-label, randomised trial. Lancet 376, 1647–1657. 21062666 10.1016/S0140-6736(10)61924-1 PMC3033534

[ref50] Dondorp A. M., Yeung S., White L., Nguon C., Day N. P., Socheat D. and von Seidlein L. (2010 *b*). Artemisinin resistance: current status and scenarios for containment. Nature Reviews Microbiology 8, 272–280. 20208550 10.1038/nrmicro2331

[ref51] Dubey J. P. (1977). Toxoplasma, Hammondia, Besnotia, Sarcocystis, and other cyst-forming coccidia of man and animals In Parasitic Protozoa (ed. Kreier J. P.), pp. 101–237. Academic Press, New York.

[ref52] Dumonteil E. (2007). DNA vaccines against protozoan parasites: advances and challenges. Journal of Biomedicine and Biotechnology 2007, 11. 10.1155/2007/90520 PMC1940056 17710244

[ref53] Duncan S. M., Myburgh E., Philipon C., Brown E., Meissner M., Brewer J. and Mottram J. C. (2016). Conditional gene deletion with DiCre demonstrates an essential role for CRK3 in *Leishmania mexicana* cell cycle regulation. Molecular Microbiology 100, 931–944. 26991545 10.1111/mmi.13375 PMC4913733

[ref54] Eyles D. E. and Coleman N. (1953). Antibiotics in the treatment of toxoplasmosis. American Journal of Tropical Medicine and Hygiene 2, 64–69. 13007923 10.4269/ajtmh.1953.2.64

[ref55] Figueiredo J. M., Rodrigues D. C., Silva R. C. M. C., Koeller C. M., Jiang J. C., Jazwinski S. M., Previato J. O., Mendonça-Previato L., Ürményi T. P. and Heise N. (2012). Molecular and functional characterization of the ceramide synthase from *Trypanosoma cruzi*. Molecular and Biochemical Parasitology 182, 62–74. 22226824 10.1016/j.molbiopara.2011.12.006 PMC3551351

[ref56] Florentino P. T. V., Real F., Bonfim-Melo A., Orikaza C. M., Ferreira E. R., Pessoa C. C., Lima B. R., Sasso G. R. S. and Mortara R. A. (2014). An historical perspective on how advances in microscopic imaging contributed to understanding the *Leishmania* spp. and *Trypanosoma cruzi* host-parasite relationship. BioMed Research International 2014, 16. 10.1155/2014/565291 PMC4022312 24877115

[ref57] Fridberg A., Olson C. L., Nakayasu E. S., Tyler K. M., Almeida I. C. and Engman D. M. (2008). Sphingolipid synthesis is necessary for kinetoplast segregation and cytokinesis in *Trypanosoma brucei*. Journal of Cell Science 121, 522–535. 18230649 10.1242/jcs.016741

[ref58] Futerman A. H. and Hannun Y. A. (2004). The complex life of simple sphingolipids. EMBO Reports 5, 777–782. 15289826 10.1038/sj.embor.7400208 PMC1299119

[ref59] Gamo F.-J., Sanz L. M., Vidal J., de Cozar C., Alvarez E., Lavandera J.-L., Vanderwall D. E., Green D. V. S., Kumar V., Hasan S., Brown J. R., Peishoff C. E., Cardon L. R. and Garcia-Bustos J. F. (2010). Thousands of chemical starting points for antimalarial lead identification. Nature 465, 305–310. 20485427 10.1038/nature09107

[ref60] Georgopapadakou N. H. (2000). Antifungals targeted to sphingolipid synthesis: focus on inositol phosphorylceramide synthase. Expert Opinion on Investigational Drugs 9, 1787–1796. 11060777 10.1517/13543784.9.8.1787

[ref61] Gerold P. and Schwarz R. T. (2001). Biosynthesis of glycosphingolipids de-novo by the human malaria parasite *Plasmodium falciparum*. Molecular and Biochemical Parasitology 112, 29–37. 11166384 10.1016/s0166-6851(00)00336-4

[ref62] Ghosh S., Bhattacharyya S., Das S., Raha S., Maulik N., Das D. K., Roy S. and Majumdar S. (2001). Generation of ceramide in murine macrophages infected with *Leishmania donovani* alters macrophage signaling events and aids intracellular parasitic survival. Molecular and Cellular Biochemistry 223, 47–60. 11681721 10.1023/a:1017996609928

[ref63] Ghosh S., Bhattacharyya S., Sirkar M., Sa G. S., Das T., Majumdar D., Roy S. and Majumdar S. (2002). *Leishmania donovani* suppresses activated protein 1 and NF-kappaB activation in host macrophages via ceramide generation: involvement of extracellular signal-regulated kinase. Infection and Immunity 70, 6828–6838. 12438359 10.1128/IAI.70.12.6828-6838.2002 PMC133095

[ref64] Gibaud S. and Jaouen G. (2010). Arsenic-based drugs: from Fowler's solution to modern anticancer chemotherapy In Medicinal Organometallic Chemistry (ed. Jaouen G. and Metzler-Nolte N.), pp. 1–20. Springer Berlin Heidelberg, Berlin, Heidelberg.

[ref65] Greif G., Harder A. and Haberkorn A. (2001). Chemotherapeutic approaches to protozoa: Coccidiae – current level of knowledge and outlook. Parasitology Research 87, 973–975. 11728025 10.1007/s004360100403

[ref66] Guillas I., Kirchman P. A., Chuard R., Pfefferli M., Jiang J. C., Jazwinski S. M. and Conzelmann A. (2001). C26-CoA-dependent ceramide synthesis of *Saccharomyces cerevisiae* is operated by Lag1p and Lac1p. Embo Journal 20, 2655–2665. 11387200 10.1093/emboj/20.11.2655 PMC125493

[ref67] Gurr M. I., Harwood J. L. and Frayn K. N. (2002). Lipid Biochemistry: An Introduction, 5th edn. Blackwell Science Ltd, Oxford, UK.

[ref68] Haberkant P. and Holthuis J. C. M. (2014). Fat & fabulous: Bifunctional lipids in the spotlight. Biochimica Et Biophysica Acta-Molecular and Cell Biology of Lipids 1841, 1022–1030. 10.1016/j.bbalip.2014.01.003 24440797

[ref69] Hallinan T. C. (1953). Drug resistance in malaria. British Medical Journal 2, 135–136. 13059413 10.1136/bmj.2.4828.135 PMC2028976

[ref70] Han G., Gable K., Yan L., Natarajan M., Krishnamurthy J., Gupta S. D., Borovitskaya A., Harmon J. M. and Dunn T. M. (2004). The topology of the Lcb1p subunit of yeast serine palmitoyltransferase. Journal of Biological Chemistry 279, 53707–53716. 15485854 10.1074/jbc.M410014200

[ref71] Hanada K. (2003). Serine palmitoyltransferase, a key enzyme of sphingolipid metabolism. Biochim Biophys Acta 1632, 16–30. 12782147 10.1016/s1388-1981(03)00059-3

[ref72] Hannun Y. A. and Obeid L. M. (2008). Principles of bioactive lipid signalling: lessons from sphingolipids. Nature Reviews Molecular Cell Biology 9, 139–150. 18216770 10.1038/nrm2329

[ref73] Harris G. H., Shafiee A., Cabello M. A., Curotto J. E., Genilloud O., Goklen K. E., Kurtz M. B., Rosenbach M., Salmon P. M., Thornton R. A., Zink D. L. and Mandala S. M. (1998). Inhibition of fungal sphingolipid biosynthesis by rustmicin, galbonolide B and their new 21-hydroxy analogs. Journal of Antibiotics 51, 837–844. 9820234 10.7164/antibiotics.51.837

[ref74] Hashidaokado T., Ogawa A., Endo M., Takesako K. and Kato I. (1995). Cloning and characterization of a gene conferring resistance to the antifungal antibiotic aureobasidin-A (R106-I) in yeast. FASEB Journal 9, A1371–A1371.

[ref75] Heidler S. A. and Radding J. A. (2000). Inositol phosphoryl transferases from human pathogenic fungi. Biochimica Et Biophysica Acta-Molecular Basis of Disease 1500, 147–152. 10.1016/s0925-4439(99)00097-6 10564728

[ref76] Heung L. J., Luberto C. and Del Poeta M. (2006). Role of sphingolipids in microbial pathogenesis. Infection and Immunity 74, 28–39. 16368954 10.1128/IAI.74.1.28-39.2006 PMC1346627

[ref77] Hojjati M. R., Li Z. and Jiang X.-C. (2005). Serine palmitoyl-CoA transferase (SPT) deficiency and sphingolipid levels in mice. Biochimica et Biophysica Acta *(*BBA*) –* Molecular and Cell Biology of Lipids 1737, 44–51. 16216550 10.1016/j.bbalip.2005.08.006

[ref78] Holthuis J. C. M. and Menon A. K. (2014). Lipid landscapes and pipelines in membrane homeostasis. Nature 510, 48–57. 24899304 10.1038/nature13474

[ref79] Holthuis J. C. M., Tafesse F. G. and Ternes P. (2006). The multigenic sphingomyelin synthase family. Journal of Biological Chemistry 281, 29421–29425. 16905542 10.1074/jbc.R600021200

[ref80] Hsu F.-F., Turk J., Zhang K. and Beverley S. M. (2007). Characterization of Inositol Phosphorylceramides from Leishmania major by Tandem Mass Spectrometry with Electrospray Ionization. Journal of the American Society for Mass Spectrometry 18, 1591–1604. 17627842 10.1016/j.jasms.2007.05.017 PMC2065762

[ref81] Huff J. (2015). The Airyscan detector from ZEISS: confocal imaging with improved signal-to-noise ratio and super-resolution. Nature Methods 12, i–ii.

[ref82] Huitema K., van den Dikkenberg J., Brouwers J. and Holthuis J. C. M. (2004). Identification of a family of animal sphingomyelin synthases. Embo Journal 23, 33–44. 14685263 10.1038/sj.emboj.7600034 PMC1271672

[ref83] Huwiler A., Kolter T., Pfeilschifter J. and Sandhoff K. (2000). Physiology and pathophysiology of sphingolipid metabolism and signaling. *Biochimica et Biophysica Acta* (*BBA*) *– Molecular and Cell Biology of Lipids* 1485, 63–99. 10832090 10.1016/s1388-1981(00)00042-1

[ref84] Ikai K., Takesako K., Shiomi K., Moriguchi M., Umeda Y., Yamamoto J., Kato I. and Naganawa H. (1991). Structure of aureobasidin-A. Journal of Antibiotics 44, 925–933. 1938614 10.7164/antibiotics.44.925

[ref85] Ikushiro H., Hayashi H. and Kagamiyama H. (2001). A water-soluble homodimeric serine palmitoyltransferase from *Sphingomonas paucimobilis* EY2395T strain. Purification, characterization, cloning, and overproduction. Journal of Biological Chemistry 276, 18249–18256. 11279212 10.1074/jbc.M101550200

[ref86] Innes E. A., Bartley P. M., Rocchi M., Benavidas-Silvan J., Burrells A., Hotchkiss E., Chianini F., Canton G. and Katzer F. (2011). Developing vaccines to control protozoan parasites in ruminants: dead or alive? Veterinary Parasitology, 180, 155–163. 21680094 10.1016/j.vetpar.2011.05.036

[ref87] Jimenez-Ruiz E., Wong E. H., Pall G. S. and Meissner M. (2014). Advantages and disadvantages of conditional systems for characterization of essential genes in *Toxoplasma gondii*. Parasitology 141, 1390–1398. 24926834 10.1017/S0031182014000559

[ref88] Kedzierski L., Sakthianandeswaren A., Curtis J. M., Andrews P. C., Junk P. C. and Kedzierska K. (2009). Leishmaniasis: current treatment and prospects for new drugs and vaccines. Current Medicinal Chemistry 16, 599–614. 19199925 10.2174/092986709787458489

[ref89] King L. (2011). The Causes and Impacts of Neglected Tropical and Zoonotic Diseases: Opportunities for Integrated Intervention Strategies. National Academies Press, Washington, DC, USA. 21977543

[ref90] Koeller C. M. and Heise N. (2011). The sphingolipid biosynthetic pathway is a potential target for chemotherapy against Chagas disease. Enzyme Research 2011, 13. 10.4061/2011/648159 PMC3092604 21603271

[ref91] Kol M., Panatala R., Nordmann M., Swart L., Van Suijlekom L., Cabukusta B., Hilderink A., Gabrietz T., Mina J. G., Somerharju P., Korneev S., Tafesse F. G. and Holthuis J. C. (2017). Switching head group selectivity in mammalian sphingolipid biosynthesis by active-site-engineering of sphingomyelin synthases. Journal of Lipid Research 58, 962–973. 28336574 10.1194/jlr.M076133 PMC5408615

[ref92] Kudo N., Kumagai K., Matsubara R., Kobayashi S., Hanada K., Wakatsuki S. and Kato R. (2010). Crystal structures of the CERT START domain with inhibitors provide insights into the mechanism of ceramide transfer. Journal of Molecular Biology 396, 245–251. 20036255 10.1016/j.jmb.2009.12.029

[ref93] Kumagai K., Yasuda S., Okemoto K., Nishijima M., Kobayashi S. and Hanada K. (2005). CERT mediates intermembrane transfer of various molecular species of ceramides. Journal of Biological Chemistry 280, 6488–6495. 15596449 10.1074/jbc.M409290200

[ref94] Kuris A. M. (2012). The global burden of human parasites: who and where are they? How are they transmitted? Journal of Parasitology 98, 1056–1064. 23016827 10.1645/12-90.1

[ref95] Labaied M., Dagan A., Dellinger M., Gèze M., Egée S., Thomas S. L., Wang C., Gatt S. and Grellier P. (2004). Anti-Plasmodium activity of ceramide analogs. Malaria Journal 3, 49. 15588325 10.1186/1475-2875-3-49 PMC539285

[ref96] Lahari S. and Futerman A. H. (2007). The metabolism and function of sphingolipids and glycosphingolipids. Cellular and Molecular Life Sciences 64, 2270–2284. 17558466 10.1007/s00018-007-7076-0 PMC11136246

[ref97] Layre E. and Moody D. B. (2013). Lipidomic profiling of model organisms and the world's major pathogens. Biochimie 95, 109–115. 22971440 10.1016/j.biochi.2012.08.012 PMC3988492

[ref98] Levine T. P., Wiggins C. A. R. and Munro S. (2000). Inositol phosphorylceramide synthase is located in the Golgi apparatus of *Saccharomyces cerevisiae*. Molecular Biology of the Cell 11, 2267–2281. 10888667 10.1091/mbc.11.7.2267 PMC14918

[ref99] Levy M. and Futerman A. H. (2010). Mammalian ceramide synthases. IUBMB Life 62, 347–356. 20222015 10.1002/iub.319 PMC2858252

[ref100] Lowther J., Naismith J. H., Dunn T. M. and Campopiano D. J. (2012). Structural, mechanistic and regulatory studies of serine palmitoyltransferase. Biochemical Society Transactions 40, 547–554. 22616865 10.1042/BST20110769

[ref101] Magee T., Prinen N., Alder J., Pagakis S. N. and Parmryd I. (2002). Lipid rafts: cell surface platforms for T cell signaling. Biological Research 35, 127–131. 12415729 10.4067/s0716-97602002000200003

[ref102] Mandala S. M., Thornton R. A., Rosenbach M., Milligan J., Garcia-Calvo M., Bull H. G. and Kurtz M. B. (1997). Khafrefungin, a novel inhibitor of sphingolipid synthesis. Journal of Biological Chemistry 272, 32709–32714. 9405490 10.1074/jbc.272.51.32709

[ref103] Mandala S. M., Thornton R. A., Milligan J., Rosenbach M., Garcia-Calvo M., Bull H. G., Harris G., Abruzzo G. K., Flattery A. M., Gill C. J., Bartizal K., Dreikorn S. and Kurtz M. B. (1998). Rustmicin, a potent antifungal agent, inhibits sphingolipid synthesis at inositol phosphoceramide synthase. Journal of Biological Chemistry 273, 14942–14949. 9614099 10.1074/jbc.273.24.14942

[ref104] Marechal E., Riou M., Kerboeuf D., Beugnet F., Chaminade P. and Loiseau P. M. (2011). Membrane lipidomics for the discovery of new antiparasitic drug targets. Trends in Parasitology 27, 496–504. 21862412 10.1016/j.pt.2011.07.002

[ref105] McAllister M. M. (2014). Successful vaccines for naturally occurring protozoal diseases of animals should guide human vaccine research. A review of protozoal vaccines and their designs. Parasitology 141, 624–640. 24476952 10.1017/S0031182013002060 PMC3961066

[ref106] Meissner M., Schlüter D. and Soldati D. (2002). Role of *Toxoplasma gondii* Myosin A in powering parasite gliding and host cell invasion. Science 298, 837–840. 12399593 10.1126/science.1074553

[ref107] Menard D. and Dondorp A. (2017). Antimalarial drug resistance: a threat to malaria elimination. Cold Spring Harbor Perspectives in Medicine, 1–25. doi: 10.1101/cshperspect.a025619. PMC5495053 28289248

[ref108] Merrill A. H. and Sandhoff K. (2002). Sphingolipids: metabolism and cell signalling In Biochemistry of Lipids, Lipoproteins and Membranes, Vol. 36, 4th edn. (ed. Vance D. E., and Vance J. E.), pp. 373–407. Elsevier Science, Amsterdam.

[ref109] Metzler D. E. (2003). Biochemistry; The Chemical Reactions of Living Cells, 2nd edn. Elsevier Academic Press, San Diego, CA, USA

[ref110] Mina J. G., Pan S. Y., Wansadhipathi N. K., Bruce C. R., Shams-Eldin H., Schwarz R. T., Steel P. G. and Denny P. W. (2009). The *Trypanosoma brucei* sphingolipid synthase, an essential enzyme and drug target. Molecular and Biochemical Parasitology 168, 16–23. 19545591 10.1016/j.molbiopara.2009.06.002

[ref111] Mina J. G., Mosely J. A., Ali H. Z., Shams-Eldin H., Schwarz R. T., Steel P. G. and Denny P. W. (2010). A plate-based assay system for analyses and screening of the *Leishmania major* inositol phosphorylceramide synthase. International Journal of Biochemistry & Cell Biology 42, 1553–1561. 20561598 10.1016/j.biocel.2010.06.008

[ref192] Mina J. G., Thye J. K., Alqaisi A. Q. I., Bird L. E., Dods R. H., Groftehauge M. K., Mosely J. A., Pratt S., Shams-Eldin H., Schwarz R. T., Pohl E. and Denny P. W. (2017). Functional and phylogenetic evidence of a bacterial origin for the first enzyme in sphingolipid biosynthesis in a phylum of eukaryotic protozoan parasites. Journal of Biological Chemistry, in press. doi: 10.1074/jbc.M117.792374. PMC5519370 28578314

[ref112] Montoya J. G. and Remington J. S. (2008). Management of *Toxoplasma gondii* infection during pregnancy. Clinical Infectious Diseases 47, 554–566. 18624630 10.1086/590149

[ref113] Murcia L., Carrilero B., Munoz-Davila M. J., Thomas M. C., López M. C. and Segovia M. (2013). Risk factors and primary prevention of congenital Chagas disease in a nonendemic country. Clinical Infectious Diseases 56, 496–502. 23097582 10.1093/cid/cis910

[ref114] Nagiec M. M., Baltisberger J. A., Wells G. B., Lester R. L. and Dickson R. C. (1994). The LCB2 gene of Saccharomyces and the related LCB1 gene encode subunits of serine palmitoyltransferase, the initial enzyme in sphingolipid synthesis. Proceedings of the National Academy of Sciences of the United States of America 91, 7899–7902. 8058731 10.1073/pnas.91.17.7899 PMC44511

[ref115] Nagiec M. M., Nagiec E. E., Baltisberger J. A., Wells G. B., Lester R. L. and Dickson R. C. (1997). Sphingolipid synthesis as a target for antifungal drugs. Journal of Biological Chemistry 272, 9809–9817. 9092515 10.1074/jbc.272.15.9809

[ref116] Newton P. N., Caillet C. and Guerin P. J. (2016). A link between poor quality antimalarials and malaria drug resistance? Expert Review of Anti-Infective Therapy 14, 531–533. 27187060 10.1080/14787210.2016.1187560

[ref117] Norcliffe J. L., Alvarez-Ruiz E., Martin-Plaza J. J., Steel P. G. and Denny P. W. (2014). The utility of yeast as a tool for cell-based, target-directed high-throughput screening. Parasitology 141, 8–16. 23611102 10.1017/S0031182013000425

[ref118] Ohanian J. and Ohanian V. (2001). Sphingolipids in mammalian cell signalling. Cellular and Molecular Life Sciences 58, 2053–2068. 11814056 10.1007/PL00000836 PMC11337328

[ref119] Ohnuki T., Yano T., Ono Y., Kozuma S., Suzuki T., Ogawa Y. and Takatsu T. (2009). Haplofungins, novel inositol phosphorylceramide synthase inhibitors, from *Lauriomyces bellulus* SANK 26899 I. Taxonomy, fermentation, isolation and biological activities. Journal of Antibiotics 62, 545–549. 19644518 10.1038/ja.2009.72

[ref120] Opremcak E. M., Scales D. K. and Sharpe M. R. (1992). Trimethoprim-sulfamethoxazole therapy for ocular toxoplasmosis. Ophthalmology 99, 920–925. 1630782 10.1016/s0161-6420(92)31873-1

[ref121] Pal R. (2015). Phase modulation nanoscopy: a simple approach to enhanced optical resolution. Faraday Discussions 177, 507–515. 25612293 10.1039/c4fd00158c

[ref122] Pankova-Kholmyansky I., Dagan A., Gold D., Zaslavsky Z., Skutelsky E., Gatt S. and Flescher E. (2003). Ceramide mediates growth inhibition of the *Plasmodium falciparum* parasite. Cellular and Molecular Life Science 60, 577–587. 10.1007/s000180300049 PMC11138575 12737317

[ref123] Paquet C. and Yudin M. H. (2013). Toxoplasmosis in pregnancy: prevention, screening, and treatment. Journal of Obstetrics and Gynaecology Canada 35, 78–81. 23343802 10.1016/s1701-2163(15)31053-7

[ref124] Parija S. C. (2016). Drug resistance in malaria: a predicament. Tropical Parasitology 6, 1. 26998428 10.4103/2229-5070.175022 PMC4778178

[ref125] Park J. W., Park W. J. and Futerman A. H. (2014). Ceramide synthases as potential targets for therapeutic intervention in human diseases. Biochim Biophys Acta 1841, 671–681. 24021978 10.1016/j.bbalip.2013.08.019

[ref126] Pata M. O., Hannun Y. A. and Ng C. K.-Y. (2010). Plant sphingolipids: decoding the enigma of the Sphinx. New Phytologist 185, 611–630. 20028469 10.1111/j.1469-8137.2009.03123.x PMC2848707

[ref127] Patnaik P. K., Field M. C., Menon A. K., Cross G. A., Yee M. C. and Butikofer P. (1993). Molecular species analysis of phospholipids from *Trypanosoma brucei* bloodstream and procyclic forms. Molecular and Biochemical Parasitology 58, 97–105. 8459838 10.1016/0166-6851(93)90094-e

[ref128] Pettus B. J., Chalfant C. E. and Hannun Y. A. (2002). Ceramide in apoptosis: an overview and current perspectives. Biochimica et Biophysica Acta *(*BBA*) –* Molecular and Cell Biology of Lipids 1585, 114–125. 12531544 10.1016/s1388-1981(02)00331-1

[ref129] Pewzner-Jung Y., Ben-Dor S. and Futerman A. H. (2006). When do Lasses (longevity assurance genes) become CerS (ceramide synthases)?: Insights into the regulation of ceramide synthesis. Journal of Biological Chemistry 281, 25001–25005. 16793762 10.1074/jbc.R600010200

[ref130] Pieperhoff M. S., Pall G. S., Jimenez-Ruiz E., Das S., Melatti C., Gow M., Wong E. H., Heng J., Muller S., Blackman M. J. and Meissner M. (2015). Conditional U1 gene silencing in *Toxoplasma gondii*. Plos ONE 10, 24. 10.1371/journal.pone.0130356 PMC4474610 26090798

[ref131] Pierce S. K. (2002). Lipid rafts and B-cell activation. Nature Reviews Immunology 2, 96–105. 10.1038/nri726 11910900

[ref132] Pratt S., Wansadhipathi-Kannangara N. K., Bruce C. R., Mina J. G., Shams-Eldin H., Casas J., Hanada K., Schwarz R. T., Sonda S. and Denny P. W. (2013). Sphingolipid synthesis and scavenging in the intracellular apicomplexan parasite, *Toxoplasma gondii*. Molecular and Biochemical Parasitology 187, 43–51. 23246819 10.1016/j.molbiopara.2012.11.007 PMC3629565

[ref133] Pruett S. T., Bushnev A., Hagedorn K., Adiga M., Haynes C. A., Sullards M. C., Liotta D. C. and Merrill A. H. (2008). Thematic review series: sphingolipids – biodiversity of sphingoid bases (‘sphingosines’) and related amino alcohols. Journal of Lipid Research 49, 1621–1639. 18499644 10.1194/jlr.R800012-JLR200 PMC2444003

[ref134] Quiñones W., Urbina J. A., Dubourdieu M. and Luis Concepción J. (2004). The glycosome membrane of *Trypanosoma cruzi* epimastigotes: protein and lipid composition. Experimental Parasitology 106, 135–149. 15172221 10.1016/j.exppara.2004.03.006

[ref135] Raas-Rothschild A., Pankova-Kholmyansky I., Kacher Y. and Futerman A. H. (2004). Glycosphingolipidoses: beyond the enzymatic defect. Glycoconjugate Journal 21, 295–304. 15514478 10.1023/B:GLYC.0000046272.38480.ef

[ref136] Ramakrishnan S., Serricchio M., Striepen B. and Bütikofer P. (2013). Lipid synthesis in protozoan parasites: a comparison between kinetoplastids and apicomplexans. Progress in Lipid Research 52, 488–512. 23827884 10.1016/j.plipres.2013.06.003 PMC3830643

[ref137] Raman M. C., Johnson K. A., Yard B. A., Lowther J., Carter L. G., Naismith J. H. and Campopiano D. J. (2009). The external aldimine form of serine palmitoyltransferase: structural, kinetic, and spectroscopic analysis of the wild-type enzyme and HSAN1 mutant mimics. Journal of Biological Chemistry 284, 17328–17339. 19376777 10.1074/jbc.M109.008680 PMC2719368

[ref138] Ramaprasad A., Mourier T., Naeem R., Malas T. B., Moussa E., Panigrahi A., Vermont S. J., Otto T. D., Wastling J. and Pain A. (2015). Comprehensive evaluation of *Toxoplasma gondii* VEG and *Neospora caninum* LIV genomes with tachyzoite stage transcriptome and proteome defines Novel transcript features. Plos ONE 10, e0124473. 25875305 10.1371/journal.pone.0124473 PMC4395442

[ref139] Ramstedt B. and Slotte J. P. (2002). Membrane properties of sphingomyelins. Febs Letters 531, 33–37. 12401199 10.1016/s0014-5793(02)03406-3

[ref140] Rao R. P., Scheffer L., Srideshikan S. M., Parthibane V., Kosakowska-Cholody T., Masood M. A., Nagashima K., Gudla P., Lockett S., Acharya U. and Acharya J. K. (2014). Ceramide transfer protein deficiency compromises organelle function and leads to senescence in primary cells. Plos ONE 9, e92142. 24642596 10.1371/journal.pone.0092142 PMC3958450

[ref141] Richmond G. S., Gibellini F., Young S. A., Major L., Denton H., Lilley A. and Smith T. K. (2010). Lipidomic analysis of bloodstream and procyclic form *Trypanosoma brucei*. Parasitology 137, 1357–1392. 20602846 10.1017/S0031182010000715 PMC3744936

[ref142] Rieckmann K. and Cheng Q. (2002). Pyrimethamine-sulfadoxine resistance in *Plasmodium falciparum* must be delayed in Africa. Trends in Parasitology 18, 293. 12379946 10.1016/s1471-4922(02)02287-0

[ref143] Romano J. D., Sonda S., Bergbower E., Smith M. E. and Coppens I. (2013). *Toxoplasma gondii* salvages sphingolipids from the host Golgi through the rerouting of selected Rab vesicles to the parasitophorous vacuole. Molecular Biology of the Cell 24, 1974–1995. 23615442 10.1091/mbc.E12-11-0827 PMC3681701

[ref144] Rovina P., Schanzer A., Graf C., Mechtcheriakova D., Jaritz M. and Bornancin F. (2009). Subcellular localization of ceramide kinase and ceramide kinase-like protein requires interplay of their Pleckstrin Homology domain-containing N-terminal regions together with C-terminal domains. Biochimica Et Biophysica Acta-Molecular and Cell Biology of Lipids 1791, 1023–1030. 10.1016/j.bbalip.2009.05.009 19501188

[ref145] Ryan U. and Hijjawi N. (2015). New developments in Cryptosporidium research. International Journal for Parasitology 45, 367–373. 25769247 10.1016/j.ijpara.2015.01.009

[ref146] Sandmeier E., Hale T. I. and Christen P. (1994). Multiple evolutionary origin of pyridoxal-5′-phosphate-dependent amino acid decarboxylases. European Journal of Biochemistry 221, 997–1002. 8181483 10.1111/j.1432-1033.1994.tb18816.x

[ref147] Sandosham A. A., Eyles D. E. and Montgomery R. (1964). Drug-resistance in falciparum malaria in South-East Asia. Medicinal Journal of Malayasia 18, 172–183. 14157183

[ref148] Schmidt T. J., Khalid S. A., Romanha A. J., Alves T. M., Biavatti M. W., Brun R., Da Costa F. B., de Castro S. L., Ferreira V. F., de Lacerda M. V. G., Lago J. H. G., Leon L. L., Lopes N. P., Amorim R. C. D., Niehues M., Ogungbe I. V., Pohlit A. M., Scotti M. T., Setzer W. N., Soeiro M. D. C., Steindel M. and Tempone A. G. (2012). The potential of secondary metabolites from plants as drugs or leads against protozoan neglected diseases – part I. Current Medicinal Chemistry 19, 2128–2175. 22414103 10.2174/092986712800229023

[ref149] Ségui B., Andrieu-Abadie N., Jaffrézou J.-P., Benoist H. and Levade T. (2006). Sphingolipids as modulators of cancer cell death: potential therapeutic targets. Biochimica et Biophysica Acta (BBA) – Biomembranes 1758, 2104–2120. 16925980 10.1016/j.bbamem.2006.05.024

[ref150] Serpeloni M., Jimenez-Ruiz E., Vidal N. M., Kroeber C., Andenmatten N., Lemgruber L., Morking P., Pall G. S., Meissner M. and Avila A. R. (2016). UAP56 is a conserved crucial component of a divergent mRNA export pathway in *Toxoplasma gondii*. Molecular Microbiology 102, 672–689. 27542978 10.1111/mmi.13485 PMC5118106

[ref151] Sevova E. S., Goren M. A., Schwartz K. J., Hsu F. F., Turk J., Fox B. G. and Bangs J. D. (2010). Cell-free synthesis and functional characterization of sphingolipid synthases from parasitic trypanosomatid protozoa. Journal of Biological Chemistry 285, 20580–20587. 20457606 10.1074/jbc.M110.127662 PMC2898309

[ref152] Sheridan C. (2011). Industry continues dabbling with open innovation models. Nature Biotechnology 29, 1063–1065. 10.1038/nbt1211-1063a 22158347

[ref153] Sidik S. M., Huet D., Ganesan S. M., Huynh M.-H., Wang T., Nasamu A. S., Thiru P., Saeij J. P. J., Carruthers V. B., Niles J. C. and Lourido S. (2016). A genome-wide CRISPR screen in toxoplasma identifies essential apicomplexan genes. Cell 166, 1423–1435.e1412. 27594426 10.1016/j.cell.2016.08.019 PMC5017925

[ref154] Sims P. F. G. (2009). Drug Resistance in *Toxoplasma gondii* In Antimicrobial Drug Resistance: Clinical and Epidemiological Aspects (ed. Mayers D. L.), pp. 1121–1126. Humana Press, Totowa, NJ.

[ref155] Smith T. K. and Bütikofer P. (2010). Lipid metabolism in *Trypanosoma brucei*. Molecular and Biochemical Parasitology 172, 66–79. 20382188 10.1016/j.molbiopara.2010.04.001 PMC3744938

[ref156] Sonda S., Sala G., Ghidoni R., Hemphill A. and Pieters J. (2005). Inhibitory effect of Aureobasidin A on *Toxoplasma gondii*. Antimicrobial Agents and Chemotherapy 49, 1794–1801. 15855498 10.1128/AAC.49.5.1794-1801.2005 PMC1087623

[ref157] Soto J. and Berman J. (2006). Treatment of New World cutaneous leishmaniasis with miltefosine. Transactions of the Royal Society of Tropical Medicine and Hygiene 100, S34–S40. 16930649 10.1016/j.trstmh.2006.02.022

[ref158] Sow S. O., Muhsen K., Nasrin D., Blackwelder W. C., Wu Y., Farag T. H., Panchalingam S., Sur D., Zaidi A. K. M., Faruque A. S. G., Saha D., Adegbola R., Alonso P. L., Breiman R. F., Bassat Q., Tamboura B., Sanogo D., Onwuchekwa U., Manna B., Ramamurthy T., Kanungo S., Ahmed S., Qureshi S., Quadri F., Hossain A., Das S. K., Antonio M., Hossain M. J., Mandomando I., Nhampossa T., Acácio S., Omore R., Oundo J. O., Ochieng J. B., Mintz E. D., O'Reilly C. E., Berkeley L. Y., Livio S., Tennant S. M., Sommerfelt H., Nataro J. P., Ziv-Baran T., Robins-Browne R. M., Mishcherkin V., Zhang J., Liu J., Houpt E. R., Kotloff K. L. and Levine M. M. (2016). The burden of cryptosporidium diarrheal disease among children <24 months of age in Moderate/High Mortality Regions of Sub-Saharan Africa and South Asia, utilizing data from the Global Enteric Multicenter Study (GEMS). PLOS Neglected Tropical Diseases 10, e0004729. 27219054 10.1371/journal.pntd.0004729 PMC4878811

[ref159] Spassieva S., Seo J. G., Jiang J. C., Bielawski J., Alvarez-Vasquez F., Jazwinski S. M., Hannun Y. A. and Obeid L. M. (2006). Necessary role for the Lag1p motif in (dihydro)ceramide synthase activity. Journal of Biological Chemistry 281, 33931–33938. 16951403 10.1074/jbc.M608092200

[ref160] Sugi T., Kato K. and Weiss L. M. (2016). An improved method for introducing site-directed point mutation into the *Toxoplasma gondii* genome using CRISPR/Cas9. Parasitology International 65, 558–562. 27167504 10.1016/j.parint.2016.05.002 PMC5035577

[ref161] Sugimoto Y., Sakoh H. and Yamada K. (2004). IPC synthase as a useful target for antifungal drugs. Current Drug Targets Infectious Disorders 4, 311–322. 15578972 10.2174/1568005043340597

[ref162] Sunder S., Jha T. K., Thakur C. P., Engel J., Sindermann H., Fischer C., Jungle K., Bryceson A. and Berman J. (2002). Oral miltefosine for Indian visceral leishmaniasis. New England Journal of Medicine 347, 1739–1746. 12456849 10.1056/NEJMoa021556

[ref163] Sutterwala S. S., Creswell C. H., Sanyal S., Menon A. K. and Bangs J. D. (2007). De novo sphingolipid synthesis is essential for viability, but not for transport of glycosylphosphatidylinositol-anchored proteins, in African trypanosomes. Eukaryot Cell 6, 454–464. 17220466 10.1128/EC.00283-06 PMC1828920

[ref164] Sutterwala S. S., Hsu F. F., Sevova E. S., Schwartz K. J., Zhang K., Key P., Turk J., Beverley S. M. and Bangs J. D. (2008). Developmentally regulated sphingolipid synthesis in African trypanosomes. Molecular Microbiology 70, 281–296. 18699867 10.1111/j.1365-2958.2008.06393.x PMC2629665

[ref165] Tafesse F. G., Vacaru A. M., Bosma E. F., Hermansson M., Jain A., Hilderink A., Somerharju P. and Holthuis J. C. M. (2014). Sphingomyelin synthase-related protein SMSr is a suppressor of ceramide-induced mitochondrial apoptosis. Journal of Cell Science 127, 445–454. 24259670 10.1242/jcs.138933

[ref166] Takesako K., Kuroda H., Inoue T., Haruna F., Yoshikawa Y., Kato I., Uchida K., Hiratani T. and Yamaguchi H. (1993). Biological properties of aureobasidin-A, a cyclic depsipeptide antifungal antibiotic. Journal of Antibiotics 46, 1414–1420. 8226319 10.7164/antibiotics.46.1414

[ref167] Talbott C. M., Vorobyov I., Borchman D., Taylor K. G., DuPré D. B. and Yappert M. C. (2000). Conformational studies of sphingolipids by NMR spectroscopy. II. Sphingomyelin. Biochimica et Biophysica Acta (BBA) – Biomembranes 1467, 326–337. 11030591 10.1016/s0005-2736(00)00229-7

[ref168] Tanaka A. K., Valero V. B., Takahashi H. K. and Straus A. H. (2007). Inhibition of Leishmania (Leishmania) amazonensis growth and infectivity by aureobasidin A. Journal of Antimicrobial Chemotherapy 59, 487–492. 17242034 10.1093/jac/dkl518

[ref169] Tatematsu K., Tanaka Y., Sugiyama M., Sudoh M. and Mizokami M. (2011). Host sphingolipid biosynthesis is a promising therapeutic target for the inhibition of hepatitis B virus replication. Journal of Medical Virology 83, 587–593. 21328371 10.1002/jmv.21970

[ref170] Thudichum J. L. W. (1884). A Treatise on the Chemical Constitution of the Brain. Archon Books, Hamden, Conn.

[ref171] Torgerson P. R. and Macpherson C. N. L. (2011). The socioeconomic burden of parasitic zoonoses: global trends. Veterinary Parasitology 182, 79–95. 21862222 10.1016/j.vetpar.2011.07.017

[ref172] Vacaru A. M., Tafesse F. G., Ternes P., Kondylis V., Hermansson M., Brouwers J., Somerharju P., Rabouille C. and Holthuis J. C. M. (2009). Sphingomyelin synthase-related protein SMSr controls ceramide homeostasis in the ER. Journal of Cell Biology 185, 1013–1027. 19506037 10.1083/jcb.200903152 PMC2711605

[ref173] Vance D. E. and Vance J. E. (2002). Biochemistry of Lipids, Lipoproteins and Membranes, 4th ed Elsevier Science.

[ref174] Verma N. K. and Dey C. S. (2004). Possible mechanism of miltefosine-mediated death of *Leishmania donovani*. Antimicrobial Agents and Chemotherapy 48, 3010–3015. 15273114 10.1128/AAC.48.8.3010-3015.2004 PMC478494

[ref175] Wang W., Yang X., Tangchaiburana S., Ndeh R., Markham J. E., Tsegaye Y., Dunn T. M., Wang G. L., Bellizzi M., Parsons J. F., Morrissey D., Bravo J. E., Lynch D. V. and Xiao S. (2008). An inositolphosphorylceramide synthase is involved in regulation of plant programmed cell death associated with defense in Arabidopsis. Plant Cell 20, 3163–3179. 19001565 10.1105/tpc.108.060053 PMC2613663

[ref176] Ward E. N. and Pal R. (2017). Image scanning microscopy: an overview. Journal of Microscopy 266, 221–228. 28248424 10.1111/jmi.12534

[ref177] Watson D. G. (2010). The potential of mass spectrometry for the global profiling of parasite metabolomes. Parasitology 137, 1409–1423. 20025826 10.1017/S0031182009991648

[ref178] Webster J. P., Kaushik M., Bristow G. C. and McConkey G. A. (2013). *Toxoplasma gondii* infection, from predation to schizophrenia: can animal behaviour help us understand human behaviour? Journal of Experimental Biology, 216, 99–112. 23225872 10.1242/jeb.074716 PMC3515034

[ref179] Weiss B. and Stoffel W. (1997). Human and murine serine-palmitoyl-CoA transferase. European Journal of Biochemistry 249, 239–247. 9363775 10.1111/j.1432-1033.1997.00239.x

[ref180] Welti R., Mui E., Sparks A., Wernimont S., Isaac G., Kirisits M., Roth M., Roberts C. W., Botte C., Marechal E. and McLeod R. (2007). Lipidomic analysis of *Toxoplasma gondii* reveals unusual polar lipids. Biochemistry 46, 13882–13890. 17988103 10.1021/bi7011993 PMC2576749

[ref181] WHO (2004). The World Health Report 2004. Changing History. WHO, Geneva.

[ref183] WHO (2016). Malaria – Fact Sheet (Dec 2016). WHO, Media Centre, vol. 2017.

[ref184] Wuts P. G. M., Simons L. J., Metzger B. P., Sterling R. C., Slightom J. L. and Elhammer A. P. (2015). Generation of Broad-Spectrum Antifungal Drug Candidates from the Natural Product Compound Aureobasidin A. ACS Medicinal Chemistry Letters 6, 645–649. 26101567 10.1021/acsmedchemlett.5b00029 PMC4468416

[ref185] Yatsu F. M. (1971). SPHINGOLIPIDOSES. California Medicine, 114, 1–&. PMC1501855 5551302

[ref186] Young S. A., Mina J. G., Denny P. W. and Smith T. K. (2012). Sphingolipid and ceramide homeostasis: potential therapeutic targets. Biochemistry Research International 2012, 12. 10.1155/2012/248135 PMC3286894 22400113

[ref187] Zhang K., Showalter M., Revollo J., Hsu F. F., Turk J. and Beverley S. M. (2003). Sphingolipids are essential for differentiation but not growth in Leishmania. Embo Journal 22, 6016–6026. 14609948 10.1093/emboj/cdg584 PMC275442

[ref188] Zhang K., Hsu F.-F., Scott D. A., Docampo R., Turk J. and Beverley S. M. (2005). Leishmania salvage and remodelling of host sphingolipids in amastigote survival and acidocalcisome biogenesis. Molecular Microbiology 55, 1566–1578. 15720561 10.1111/j.1365-2958.2005.04493.x PMC3803142

[ref189] Zhang K., Bangs J. D. and Beverley S. M. (2010). Sphingolipids in parasitic protozoa. Advances in Experimental Medicine and Biology 688, 238–248. 20919659 10.1007/978-1-4419-6741-1_17 PMC2951629

[ref190] Zhou L. J., Xia J., Wei H. X., Liu X. J. and Peng H. J. (2017). Risk of drug resistance in *Plasmodium falciparum* malaria therapy-a systematic review and meta-analysis. Parasitology Research 116, 781–788. 28028628 10.1007/s00436-016-5353-2

[ref191] Zofou D., Nyasa R. B., Nsagha D. S., Ntie-Kang F., Meriki H. D., Assob J. C. N. and Kuete V. (2014). Control of malaria and other vector-borne protozoan diseases in the tropics: enduring challenges despite considerable progress and achievements. Infectious Diseases of Poverty 3, 1. 24401663 10.1186/2049-9957-3-1 PMC3895778

